# Efficacy evaluation of the cervical posterior “keyhole technique” in the treatment of cervical radiculopathy: A retrospective case series

**DOI:** 10.1371/journal.pone.0359633

**Published:** 2026-10-27

**Authors:** Ruohui Tang, Jin Yin, Zhixiang Zhang, Zichao Chen, Yixin Li, Zhaowen Zhou

**Affiliations:** Department of Orthopedic, Kunming First People’s Hospital, Affiliated Calmette Hospital of Kunming Medical University, Kunming, Yunnan, China; Columbia University Vagelos College of Physicians and Surgeons, UNITED STATES OF AMERICA

## Abstract

**Study design:**

Retrospective clinical study.

**Objective:**

To evaluate the efficacy of endoscopic posterior keyhole decompression for cervical radiculopathy and to investigate the correlation between decompression parameters and clinical outcomes.

**Summary of background data:**

Cervical radiculopathy traditionally requires anterior cervical discectomy and fusion (ACDF) or open posterior decompression. Although ACDF is the gold standard, it is associated with adjacent segment degeneration and implant-related complications. Minimally invasive posterior keyhole techniques aim to reduce surgical trauma; however, the optimal extent of decompression required for symptomatic relief remains unclear.

**Methods:**

Forty patients with single-level cervical radiculopathy treated via unilateral endoscopic posterior keyhole decompression (strictly limited to targeted foraminotomy and partial medial facetectomy) (2021–2024) were reviewed. Clinical outcomes were assessed using the Visual Analog Scale (VAS) and Neck Disability Index (NDI) preoperatively, at discharge, and at 6 months postoperatively. Decompression parameters were quantified via postoperative CT. Statistical analysis included paired t-tests and Pearson correlation.

**Results:**

All 40 procedures were successful. Specifically, 25 patients underwent isolated foraminotomy, and 15 received foraminotomy combined with discectomy, without any progression to interlaminar decompression or laminectomy. The mean operative time was 70.4 ± 24.7 minutes, and the mean blood loss was 11.7 ± 10.9 ml. Significant improvements in clinical scores were observed at all postoperative time points (P < 0.05). Statistical analysis revealed that neither the concurrent execution of a discectomy nor the diameter of the bony decompression window was significantly associated with the degree of postoperative symptom improvement.

**Conclusions:**

This study demonstrates that endoscopic posterior keyhole decompression is a safe and feasible technique that provides favorable short-term clinical outcomes with minimal surgical trauma. The findings strongly suggest that clinical efficacy depends on targeted neural decompression (determined by intraoperative nerve root mobility) rather than the maximal removal of disc tissue or extensive laminoforaminotomy.

## Introduction

Cervical spondylosis is a widespread degenerative disorder whose clinical manifestations vary by age, gender, and occupation, with a notable trend toward increasing prevalence in younger demographics. It is crucial to distinctively classify CSR from degenerative cervical myelopathy (DCM). As clarified by the AO Spine Knowledge Forum [1], DCM primarily involves spinal cord compression and upper motor neuron signs, whereas CSR is strictly characterized by isolated nerve root compression leading to dermatomal and myotomal deficits. While conservative management remains the primary therapeutic approach for CSR, a subset of patients requires surgical intervention due to refractory symptoms [2]. Historically, anterior cervical discectomy and fusion (ACDF) has been considered the “gold standard.” Over the past two decades, the surgical management of CSR has evolved significantly. While ACDF remains common, an increasing number of studies have demonstrated the efficacy of full-endoscopic posterior cervical foraminotomy. For instance, Ruetten S et al. [3] and Yang JS et al. [4] conducted comparative studies indicating that endoscopic approaches yield clinical outcomes comparable to conventional anterior surgery, but with the distinct advantages of reduced tissue trauma and preservation of segmental mobility. Similarly, Kim et al. [5] showed that posterior foraminotomy offers excellent long-term clinical and biomechanical results for posterolateral soft disc herniations. These foundational studies have fueled the ongoing debate between anterior and posterior approaches, highlighting the need for further refinement of endoscopic indications. However, recent literature highlights the inherent risks of the anterior approach, including dysphagia, adjacent segment degeneration (ASD), and the loss of segmental motion [6]. Consequently, posterior minimally invasive spine surgery (MISS) has gained significant traction. Specifically, the endoscopic posterior cervical foraminotomy (the “keyhole” technique) has emerged as a highly effective alternative, aiming to directly decompress the nerve root while preserving the intervertebral disc and motion segment [7–9].

Despite the technical maturation of the posterior endoscopic approach, a critical clinical debate remains unresolved: whether concurrent removal of the intervertebral disc (discectomy) is strictly necessary during the foraminotomy, or if bony decompression alone is sufficient for optimal symptomatic relief. To further evaluate the clinical efficacy of this approach and to provide preliminary insights into the necessity of adjunctive discectomy, we conducted a retrospective analysis of 40 patients with single-level cervical radiculopathy treated via the posterior keyhole technique at the First People’s Hospital of Kunming between March 2021 and December 2024.

## Materials and methods

### General data and patient selection

This study was approved by the Institutional Review Board (Ethics Committee) of Kunming First People’s Hospital (Approval No. 2025-018-01). All procedures were performed in accordance with the Declaration of Helsinki. The requirement for informed consent was waived by the Ethics Committee of Kunming First People’s Hospital due to the retrospective nature of the study. Because this is a retrospective observational study evaluating past medical records, the requirement for direct patient consent was waived by the Ethics Committee. All patient data were anonymized before analysis to protect patient privacy. The study included patients who underwent surgery between March 2021 and December 2024. For research purposes, the medical records and clinical data were accessed between 01/06/2025 and 31/08/2025.

The inclusion criteria were: (1) unilateral single-level CSR diagnosed clinically and radiographically; (2) failure of strict conservative treatment for at least 6 weeks; and (3) presence of corresponding soft disc herniation or foraminal bony stenosis. The exclusion criteria were: (1) clinical or radiographic evidence of cervical myelopathy (DCM); (2) severe cervical instability or kyphotic deformity; (3) multi-level symptomatic radiculopathy; (4) previous cervical spine surgery at the same level; and (5) active spinal infection or malignancy. The cohort consisted of 22 males and 18 females, with an average age of 56.8 ± 8.54 years. The treated levels included C4/5 (n = 1), C5/6 (n = 19), C6/7 (n = 19), and C7/T1 (n = 1). The distribution of surgical segments is illustrated in Fig 1.

**Fig 1:**
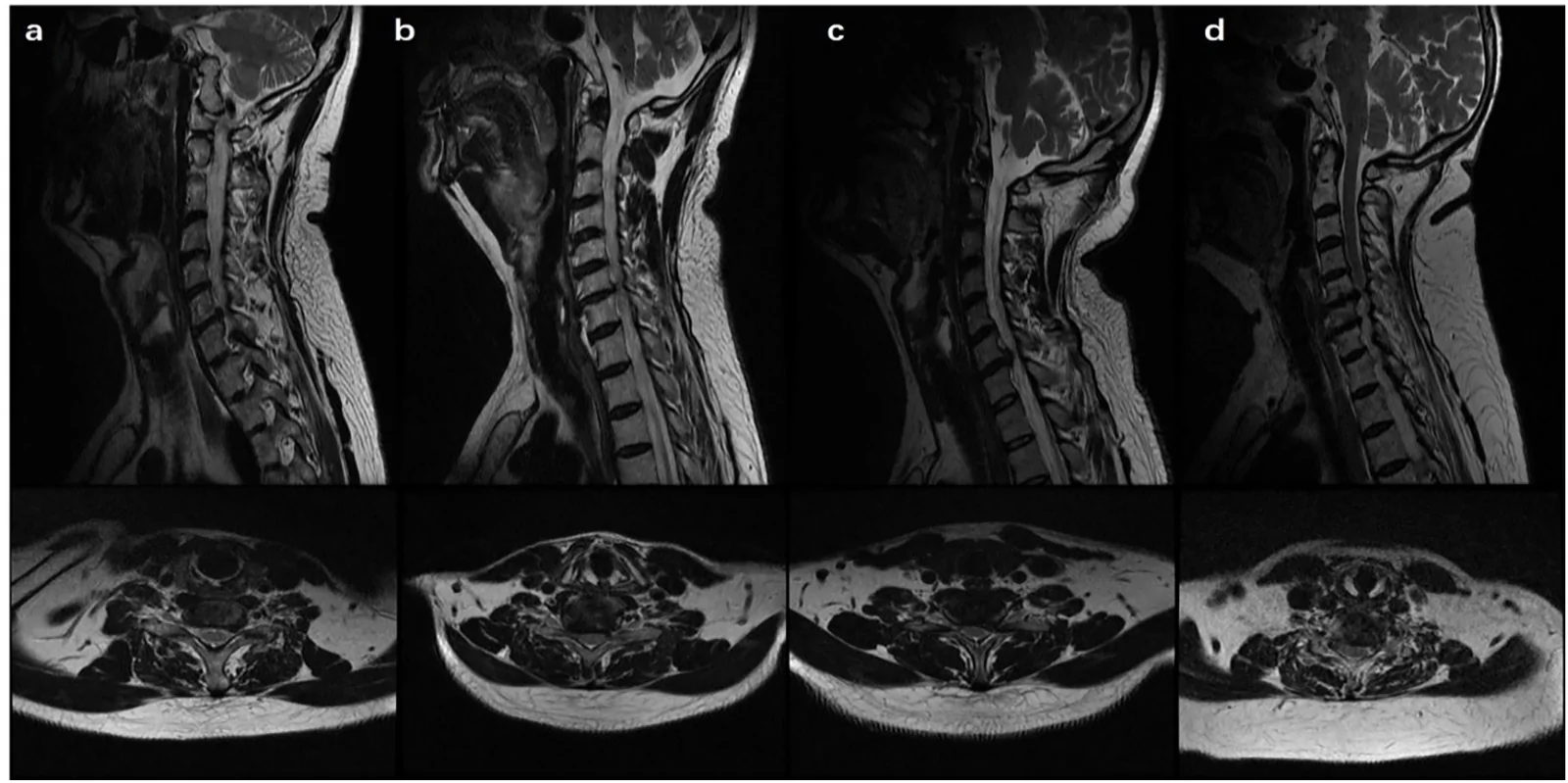
A. 52-year-old male, left-sided disc herniation at C6/7; B 66-year-old male, right-sided disc herniation at C6/7; C. 37-year-old male, left-sided disc herniation at C6/7; D. 69-year-old female, left-sided disc herniation at C5/6.

### Diagnostic criteria

The diagnosis of cervical spondylotic radiculopathy (CSR) was based on the “Expert Consensus on the Standardization of Diagnosis and Treatment of Cervical Spondylotic Radiculopathy (2015),” requiring the fulfillment of the following criteria: 1) Clinical Manifestations: Presence of typical radicular symptoms and neurological signs (e.g., sensory or motor deficits) that correspond precisely to the dermatomal or myotomal distribution of the affected cervical nerve roots. 2) Provocative Maneuvers: Positive results in the brachial plexus tension test (Eaton’s sign) or the neck compression test (Spurling’s sign). 3) Imaging Confirmation: Radiographic and CT evidence of cervical degeneration (e.g., osteophyte formation, foraminal stenosis) and MRI confirmation of nerve root compression at the symptomatic level. 4) Differential Diagnosis: Exclusion of other pathologies presenting with upper limb pain, such as rotator cuff disorders, thoracic outlet syndrome, lateral epicondylitis (tennis elbow), and organic spinal lesions (e.g., primary or metastatic tumors, spinal tuberculosis).

### Surgical method

All procedures were performed using the TH8700-031L endoscopic system. After induction of general anesthesia, patients were placed in the prone position with the head stabilized in a neutral or slightly flexed position using a headrest. The upper limbs were positioned at the sides and, when necessary, tractioned caudally and secured with adhesive tape to enhance fluoroscopic visualization of the lower cervical segments. The target level and corresponding skin projection were localized under C-arm fluoroscopy. A 1.0-cm longitudinal skin incision was made at the identified entry point. An 8-mm outer diameter working channel was introduced stepwise using a series of blunt dilators. The endoscope was then inserted and coupled with a continuous irrigation system, a light source, and a suction device. Under endoscopic visualization, soft tissues were dissected using a radiofrequency (RF) electrode and endoscopic graspers to expose the “V-point”—the anatomical junction formed by the inferior margin of the superior lamina, the superior margin of the inferior lamina, and the medial aspect of the facet joint. A high-speed diamond burr was utilized to perform a laminotomy and partial medial facetectomy. This involved resecting the inferolateral margin of the cranial lamina, the superolateral margin of the caudal lamina, and the medial portion of the facet joint. The ligamentum flavum was then removed to fully decompress the lateral thecal sac and the exiting nerve root.

It should be clearly specified that the scope of decompression in our technique was aimed exclusively at foraminotomy and partial medial facetectomy; no interlaminar decompression or laminectomy was performed. Meticulous hemostasis was maintained using the RF electrode. The decision to perform a concurrent discectomy was determined by a combination of preoperative MRI findings and intraoperative exploration. Specifically, if intraoperative assessment revealed that the posterior annulus fibrosus remained intact and the nerve root tension decreased significantly after precise microscopic bony and ligamentous decompression, the disc was not excised. We maintain that precise posterior decompression is the critical factor in relieving nerve root compression symptoms. After confirming the adequate decompression and mobility of the nerve root, the endoscope was withdrawn, and the incision was closed with interrupted sutures. In cases with concomitant disc herniation that met the aforementioned criteria, a targeted fragmentectomy was performed, followed by thermal annuloplasty using the RF electrode (Fig 2).

**Fig 2:**
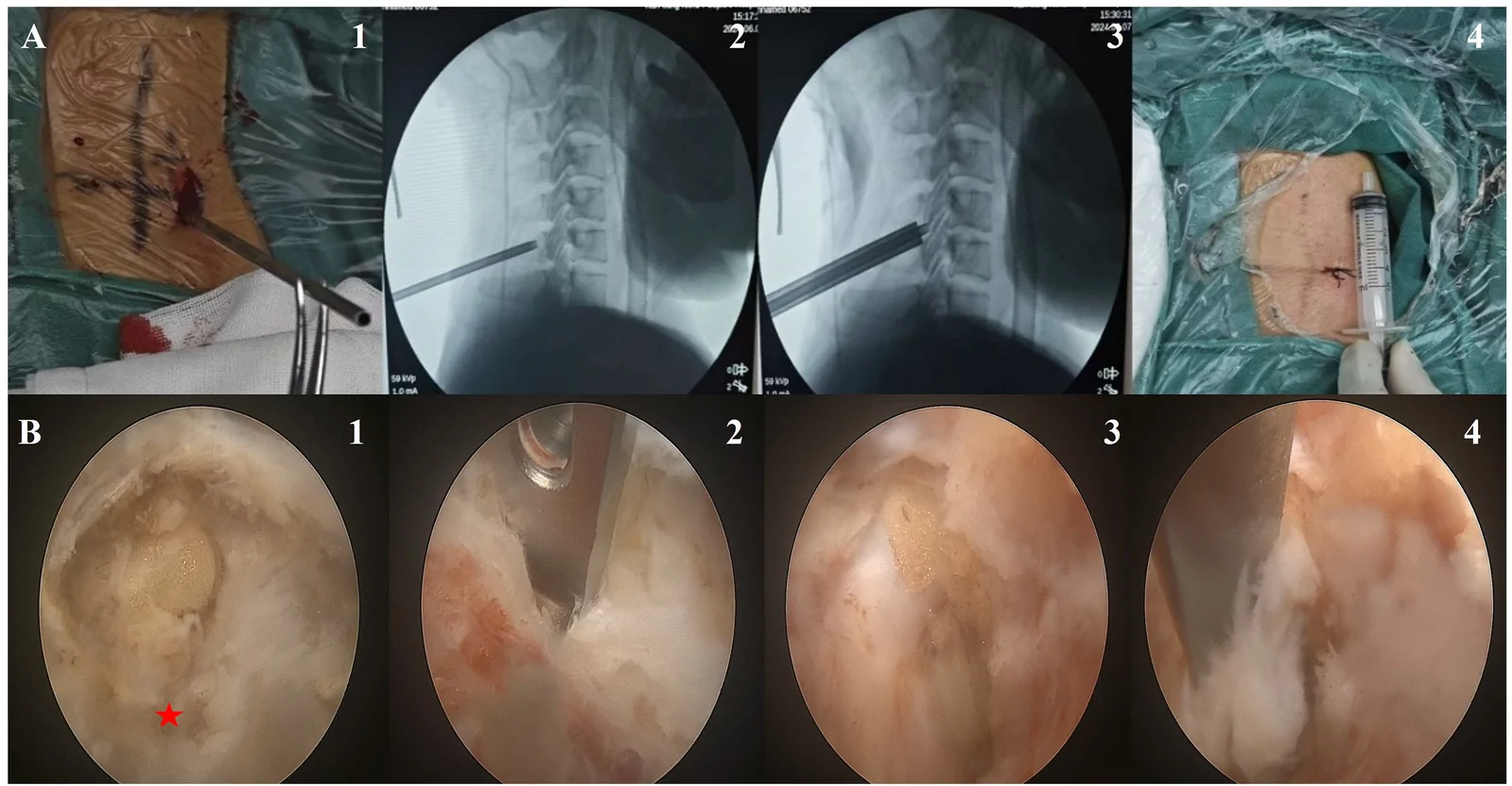
A. Lateral midline puncture positioning, intraoperative confirmation of puncture segment, establishment of working channel, and postoperative appearance with a 1.0 cm incision. **B.** Main intraoperative steps of the endoscopic procedure. **B1:** After confirming the surgical level via fluoroscopy, the working channel and endoscope are sequentially inserted. Under endoscopic visualization, the soft tissues on the surface of the lamina and lateral mass joint are cleared. The “V-point” (indicated by a star) is exposed laterally through the interlaminar space, and a high-speed burr is used to drill the bone at the V-point. **B2:** A Kerrison rongeur is used to bite off the residual thin layer of bone, further exposing the V-point. **B3:** The lateral edge of the ligamentum flavum and the dorsal membranous structure of the nerve root are exposed. After identifying the nerve root, a partial resection of the ligamentum flavum is performed to further decompress the dorsal aspect of the nerve root canal. **B4:** A nerve hook is used to gently retract the nerve root. The axilla and shoulder regions of the nerve root are carefully explored, and the herniated disc material (nucleus pulposus) is removed to achieve complete release and mobilization of the nerve root.

### Perioperative management and outcome measures

To minimize perioperative capillary hemorrhage and local inflammatory response, 0.5 g of tranexamic acid was administered intravenously 30 minutes prior to the incision, followed by an additional 0.5 g dose postoperatively. Postoperative analgesia was standardized using nonsteroidal anti-inflammatory drugs (NSAIDs). Additionally, depending on the severity of residual symptoms, adjunct therapies including osmotic diuretics, low-dose glucocorticoids, or neurotrophic agents were administered.

Additionally, local steroid injections were not utilized during the procedures. Systemic steroids were not administered routinely. Only in cases where patients experienced transient limb numbness within 24 hours postoperatively (n = 3), an intravenous dose of 60 mg methylprednisolone was administered to alleviate nerve root edema. For patients who underwent adjunctive discectomy, a soft cervical collar was recommended during physical activities for 6–8 weeks to ensure localized stability during the early healing phase. Regular outpatient follow-ups were conducted monthly to evaluate symptomatic recovery and monitor cervical stability via dynamic radiographic studies. Clinical outcomes were quantified using the Visual Analog Scale (VAS) for pain, the Japanese Orthopedic Association (JOA) score for neurological function, and the Neck Disability Index (NDI) for functional disability. The adequacy of neural decompression was assessed by comparing preoperative MRI with postoperative CT scans, specifically focusing on the dimensions of the newly created bony window and the relief of foraminal stenosis. All radiological measurements were performed independently by two spine surgeons who were not involved in the surgical procedures and were strictly blinded to the patients’ clinical outcomes. Any discrepancies were resolved by consensus.

### Statistical methods

Statistical analysis was conducted using SPSS version 22.0. Continuous variables, including clinical scores and surgical metrics, were expressed as mean ± standard deviation (SD). The normality of the data distribution was confirmed using the Shapiro-Wilk test. To compare clinical outcomes (VAS, JOA, and NDI scores) between preoperative and postoperative time points (at discharge and 6-month follow-up), the paired t-test was employed. Pearson correlation analysis was utilized to assess the relationship between the degree of improvement in clinical scores and the transverse diameter of the decompression window. A *P*-value of less than 0.05 was considered statistically significant.

## Results

### Perioperative data

All 40 procedures were completed successfully without conversion to open surgery. The mean operative time was 70.4 ± 24.7 minutes, with a mean intraoperative blood loss of 11.7 ± 10.9 mL. The average hospital stay was 6.2 ± 3.2 days, and the mean hospitalization cost was 10,706.2 ± 4,205.8 CNY. During the follow-up period, two patients experienced a recurrence of symptoms. Both patients subsequently underwent Anterior Cervical Discectomy and Fusion (ACDF) surgery and achieved satisfactory relief. Clinical outcomes, as measured by VAS, JOA, and NDI scores, demonstrated significant improvement at both discharge and the 6-month postoperative follow-up compared to preoperative baselines (all *P* < 0.05, Table 1). Specifically, the mean VAS score decreased from 3.9 ± 1.2 preoperatively to 2.2 ± 1.0 at discharge and 1.1 ± 0.8 at 6 months. The mean JOA score improved from 12.4 ± 1.6 preoperatively to 13.9 ± 1.6 and 15.6 ± 1.3 at the respective follow-up points. Furthermore, the mean NDI score significantly declined from 16.1 ± 5.3 preoperatively to 9.6 ± 3.9 at discharge and 4.9 ± 3.2 at the 6-month follow-up.

**Table 1:** VAS, JOA, and NDI scores at the preoperative, before-discharge, and 6-month postoperative assessments. Values are mean ± SD. The P values refer to paired comparisons between the preoperative score and the score at the corresponding later time point.

Time Point	VAS Score	JOA Score	NDI Score
**Preoperative**	3.9 ± 1.22	12.4 ± 1.57	16.1 ± 5.28
**Before Discharge**	2.2 ± 1.00	13.9 ± 1.61	9.6 ± 3.89
**6 Months Postoperative**	1.1 ± 0.76	15.6 ± 1.29	4.9 ± 3.16
**Preoperative/Before Discharge P-value**	1.1624 × 10^−9^	3.7307 × 10 ^−13^	2.2759 × 10^−17^
**Preoperative/6 Months Postoperative P-value**	1.3401 × 10^−15^	1.2035 × 10^−17^	5.2116 × 10 ^−19^

### Imaging findings

A subgroup of 20 patients (14 males, 6 females) underwent postoperative CT evaluation to assess the anatomical extent of decompression (Fig 3). The remaining 20 patients did not undergo postoperative CT or MRI scans. This was primarily due to rapid postoperative discharge (often on postoperative day 1 or 2) prior to scheduling the scan. Furthermore, strict health insurance payment policies and the additional financial burden led to direct patient refusal to undergo routine postoperative imaging, especially given their significant clinical symptomatic improvement. The surgical levels in this subgroup included C4/5 (n = 1), C5/6 (n = 9), and C6/7 (n = 10) (Table 2). Based on postoperative CT analysis, the mean maximum transverse diameter of the decompression window was 8.7 ± 0.93 mm. The mean lateral offset of the decompression center from the midline was 13.5 ± 1.13 mm, and the radiographic depth of the decompression area was 62.3 ± 15.50 mm (Table 3). Decompression depth was measured on axial CT slices. Notably, as the surgical trajectory was not perpendicular to the coronal plane, a systematic discrepancy exists between the measured radiographic depth and the actual intraoperative insertion depth. Pearson correlation analysis was performed to evaluate the relationship between the transverse diameter of the decompression window and clinical recovery (improvements in VAS, JOA, and NDI). No statistically significant correlations were identified (all *P* > 0.05), suggesting that clinical symptomatic relief was not significantly associated with the width of the decompression window within the range observed (Fig 4).

**Fig 3:**
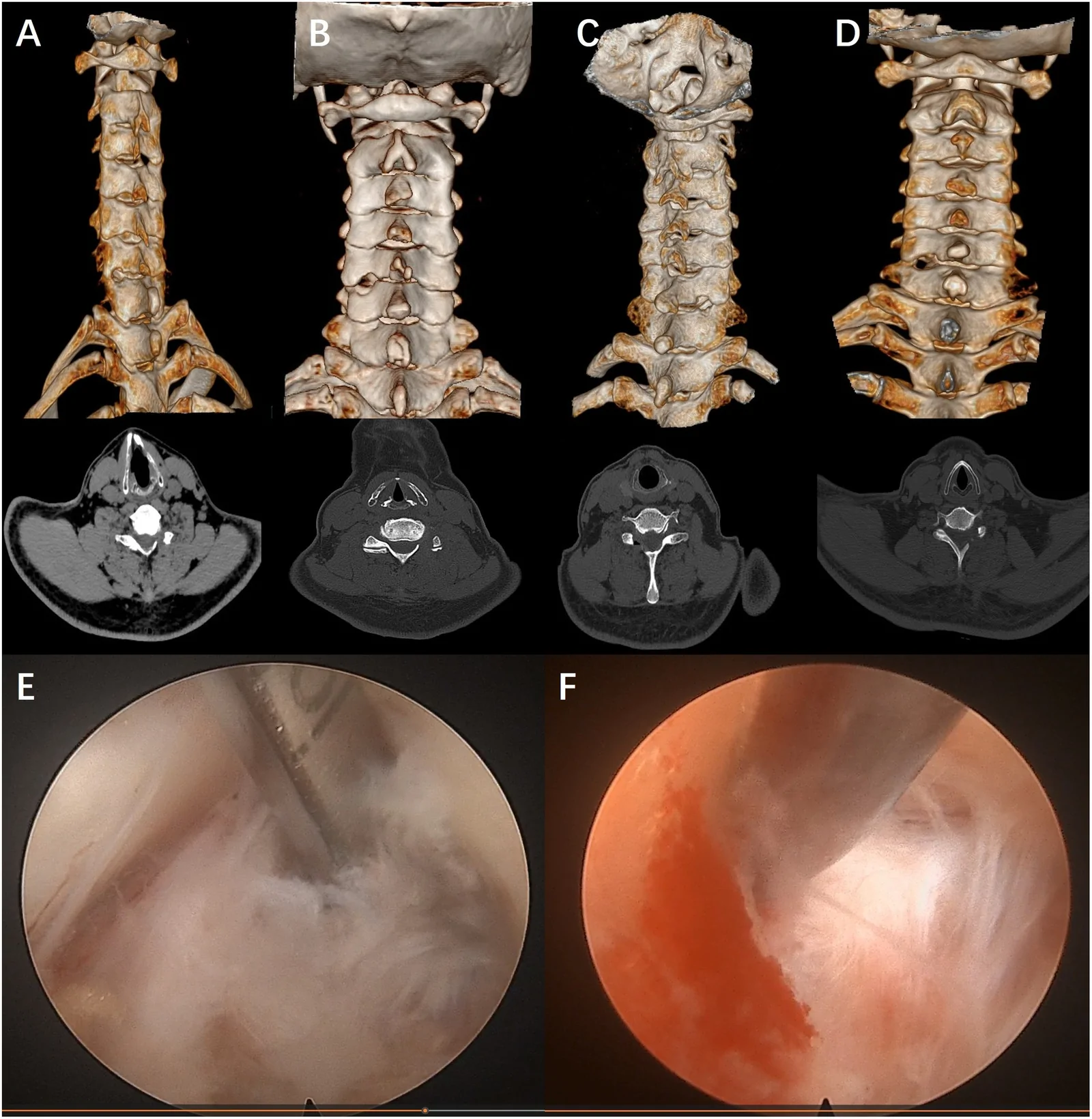
A. 46-year-old male, postoperative left-sided foraminal decompression at C6/7; B. 54-year-old female, postoperative left-sided foraminal decompression at C5/6; C. 57-year-old male, postoperative right-sided foraminal decompression at C6/7; D. 45-year-old male, left-sided foraminal decompression at C6/7. **E.** Disc herniation with annulus fibrosus rupture, concurrent removal of the herniated nucleus pulposus during transforaminal decompression. **F.** Contained disc herniation with an intact annulus fibrosus, isolated transforaminal decompression was performed.

**Table 2:** Segment distribution and segment-specific measurements of the maximum decompression transverse diameter, puncture depth, and center-point offset in the cervical spine. The number of cases is shown for each segment.

Segment	Number of Cases	Maximum Decompression Transverse Diameter (mm)	Puncture Depth (mm)	Offset of Center Point (mm)
C4/5	1	7.3	45.4	13.09
C5/6	9	8.8 ± 0.55	57.3 ± 13.3	12.9 ± 1.55
C6/7	10	9.0 ± 0.98	68.7 ± 13.8	13.9 ± 0.92

**Table 3:** Correlations between the decompression transverse diameter and VAS, JOA, and NDI scores. The shared heading above the right-hand columns denotes the decompression transverse diameter (mm).

	Decompression Transverse Diameter/mm	
**Variable**	**Correlation Coefficient *r***	**P-value**
**VAS Score**	−0.229	0.332
**JOA Score**	0.213	0.368
**NDI Score**	0.080	0.736

**Fig 4:**
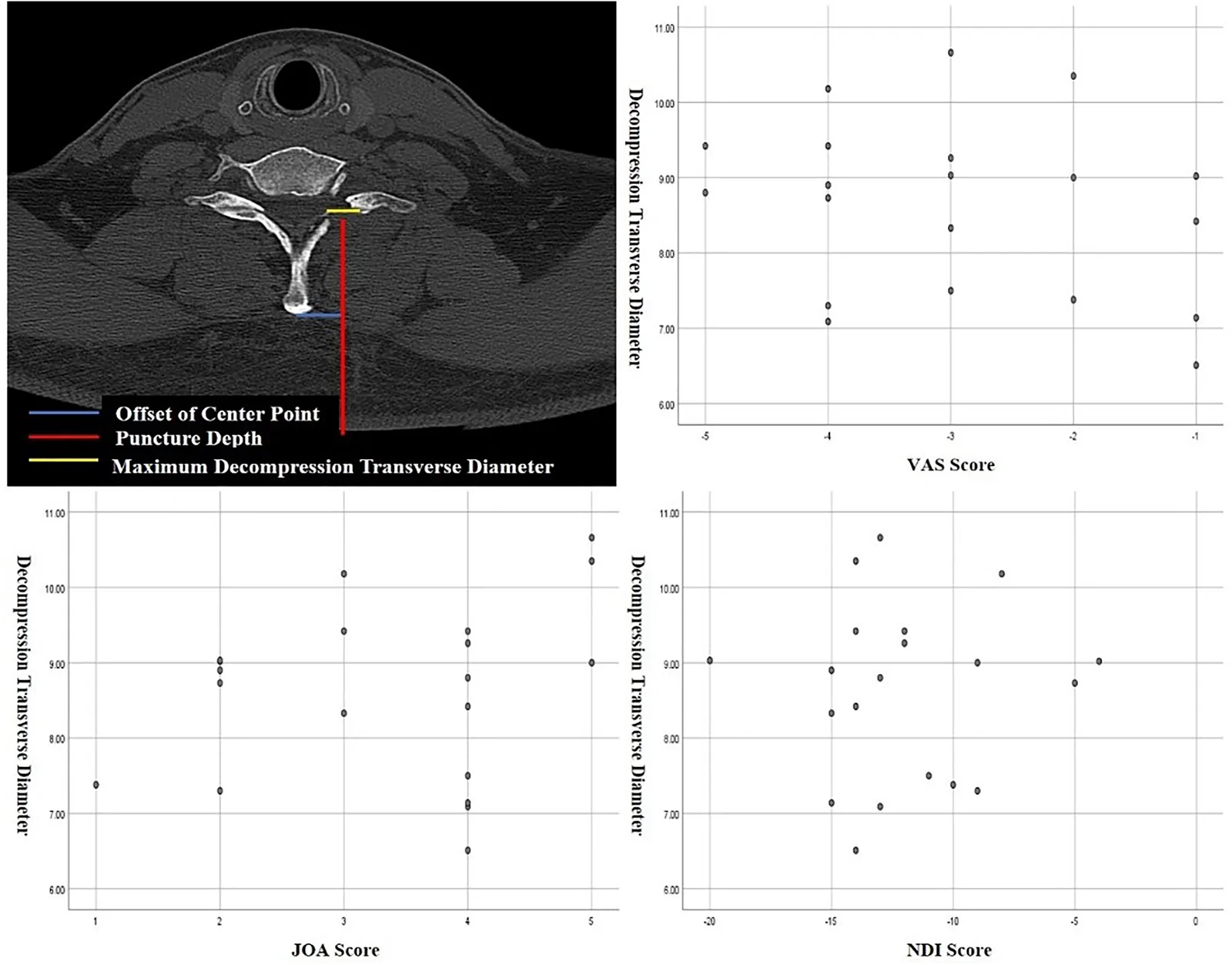
Axial CT image from postoperative follow-up. Scatter plots showing the relationship between improvements in VAS score, JOA score, NDI score, and the decompression transverse diameter.

### Quantitative evaluation

Based on preoperative pathology and surgical planning, the cohort was divided into two subgroups: 25 patients underwent isolated foraminotomy, and 15 patients underwent foraminotomy combined with discectomy (removal of the herniated nucleus pulposus). None of the patients in our cohort received interlaminar decompression or laminectomy. Intergroup analysis revealed no statistically significant differences between these two subgroups regarding operative time, intraoperative blood loss, or hospitalization costs (all *P* > 0.05). Furthermore, there were no significant differences in preoperative JOA scores, postoperative JOA scores, or the JOA recovery rate between the groups (Table 4). (JOA recovery rate = [postoperative score – preoperative score] / [17 – preoperative score] × 100%). However, given the limited sample size, these findings should be interpreted with caution and cannot definitively establish the clinical equivalence of the two strategies.

**Table 4:** Comparison of perioperative outcomes and JOA scores between decompression alone and decompression combined with nucleus pulposus removal. Values are mean ± SD. F and P values report between-group comparisons for each variable.

Group	Number of Cases	Surgical Time (min)	Blood Loss (ml)	Hospitalization Cost (CNY)	Preoperative JOA Score	Postoperative JOA Score at 6 Months	Improvement Rate
**Decompression Only**	25	69.1 ± 23.1	13.4 ± 12.7	10792.2 ± 5076.7	12.7 ± 1.40	15.6 ± 1.44	0.75 ± 0.16
**Decompression + Nucleus Pulposus Removal**	15	72.3 ± 28.4	9.1 ± 7.5	10577 ± 2734.8	11.9 ± 1.73	15.7 ± 1.05	0.68 ± 0.32
**F-value**	/	0.15	1.41	0.02	2.56	0.32	0.85
**P-value**	/	0.69	0.24	0.88	0.11	0.57	0.36

## Discussion

In this study, the significant improvements in VAS, JOA, and NDI scores observed at discharge and the 6-month follow-up underscore the feasibility and safety of the posterior “keyhole” technique for treating unilateral CSR. Traditionally, ACDF has been the gold standard for single-level cervical radiculopathy. Since Ruetten et al. [10] introduced the posterior full-endoscopic interforaminal technique in 2007, it has been discussed in the literature as a minimally invasive alternative. Meta-analyses by Sahai et al. [11] and Fang et al. [12] have reported that the posterior approach yields similar improvements in clinical scores compared to ACDF. While the rapid recovery and high patient satisfaction observed in our cohort align with these general background findings, it is important to emphasize that because our study lacks a comparison group, we cannot draw any conclusions regarding whether this technique is equivalent or superior to ACDF. It is worth noting that the average length of hospital stay in our cohort might appear longer than typically reported in outpatient settings. This duration was not driven by surgical complications or delayed postoperative recovery; rather, it reflects local hospital protocols where routine preoperative examinations and scheduling delays (1-to-3 day waiting period for inpatient MRI appointments) contribute to the overall hospitalization time.A primary focus of our study was whether the concurrent removal of the nucleus pulposus is essential during posterior decompression. Our sub-group analysis revealed no significant differences in clinical recovery or JOA improvement rates between patients who underwent isolated foraminotomy and those who received adjunctive discectomy. However, this subgroup comparison is significantly underpowered (n = 25 vs. n = 15). Therefore, the lack of statistical significance does not establish clinical equivalence between the two approaches. While our preliminary clinical observation suggests that isolated bony decompression might provide short-term symptomatic relief for certain encapsulated protrusions without the need for aggressive disc removal, this hypothesis remains speculative and requires validation in adequately powered studies.

Regarding the two cases of recurrence in our cohort, we hypothesize that posterior decompression provides limited relief for severe, progressive anterior compression. Furthermore, the progression of adjacent segment degeneration (ASD) can lead to recurrent symptoms that overlap with the initial presentation. Therefore, for patients with massive protrusions or severe ASD, preoperative counseling must emphasize these limitations to manage treatment expectations. Additionally, while we administered an intravenous dose of methylprednisolone in a small subset of patients (n = 3) who experienced transient postoperative limb numbness, systemic steroids were not used routinely. The sustained improvements observed in the overall cohort at the 6-month follow-up indicate that mechanical decompression is the primary driver of recovery.Precise anatomical localization is the cornerstone of the keyhole technique. Our CT follow-up identified the optimal decompression center at an average of 13.5 mm from the midline. The “V-point” remains the critical intraoperative landmark; however, its projection varies by segment. As noted by some scholars, the V-point moves medially relative to the dura mater in lower cervical segments, necessitating more lateral bone resection while exercising caution medially to protect the spinal cord [13].

Regarding the extent of decompression, our correlation analysis showed that clinical improvement was not significantly associated with the transverse diameter of the bone window (average 8.7–8.8 mm). While this observation aligns with the concept of “targeted decompression” (focusing on precise neural foramen enlargement rather than extensive bone removal), the current study does not have sufficient power or design strength to make definitive mechanistic claims. We cannot conclusively state that clinical efficacy depends solely on targeted neural decompression rather than wider laminoforaminotomy. To preserve vertebral stability, it is essential to retain more than 50% of the facet joint [14]. While the traditional keyhole technique is localized, more advanced approaches, such as the unilateral approach for bilateral decompression, can offer a wider decompression range when necessary [15]. Furthermore, while our study focuses on single-level pathology, the management of multilevel or bilateral pathology warrants attention. For multilevel disease, multiple targeted keyholes can be performed sequentially while preserving overall stability. For bilateral symptoms, an over-the-top approach (unilateral laminotomy for bilateral decompression) may be considered, although it requires advanced endoscopic proficiency.Intraoperative safety is further influenced by physiological factors; for instance, the irrigation pressure and temperature used during endoscopy may correlate with postoperative neck pain [13]. To mitigate risks, neurophysiological monitoring can be combined with the keyhole technique, providing the safety benefits of local anesthesia (nerve monitoring) with the patient comfort of general anesthesia [16]. Furthermore, emerging technologies like Augmented Reality (AR) are poised to revolutionize this field by providing real-time visualization of extra-canalicular structures, thereby improving the accuracy for beginners and reducing surgical risks.Despite the promising clinical outcomes, several critical limitations of this study must be acknowledged. First, the retrospective and single-center nature of the study may introduce selection bias. Second, the small sample size (N = 40) and the brief 6-month follow-up severely restrict the statistical power of the study. Specifically, the subgroup analysis (isolated foraminotomy vs. combined discectomy) lacks the statistical power required to claim true clinical “equivalence”; it merely indicates that no significant differences were observed within this limited cohort. Third, and most importantly, this study lacks a control group. Without a direct comparison to alternative and widely accepted surgical approaches—such as Anterior Cervical Discectomy and Fusion (ACDF) or traditional open posterior foraminotomy—the relative efficacy and safety of the endoscopic keyhole technique cannot be definitively established. Finally, the 6-month follow-up period is relatively short and insufficient to assess long-term outcomes. Future prospective, multicenter randomized controlled trials with larger cohorts and extended follow-up periods are essential to assess long-term durability, including late recurrence, adjacent segment disease, and delayed spinal instability, as well as to directly compare this technique with other standard treatments.

## Supporting information

S1 Data Supplementary information. (XLSX)

## Abbreviations

CT: Computed tomography
MRI: Magnetic Resonance Imaging
SD: Standard deviation
CSR: Cervical spondylotic radiculopathy
ACDF: Anterior cervical discectomy and fusion
VAS: Visual Analog Scale
JOA: Japanese Orthopedic Association
NDI: Neck Disability Index (NDI)

## References

[1] Ganau M, Holly LT, Mizuno J, Fehlings MG. Future Directions and New Technologies for the Management of Degenerative Cervical Myelopathy. Neurosurg Clin N Am. 2018;29(1):185–93. doi: 10.1016/j.nec.2017.09.006 29173432

[2] Smith GW, Robinson RA. The treatment of certain cervical-spine disorders by anterior removal of the intervertebral disc and interbody fusion. J Bone Joint Surg Am. 1958;40(3):607–24. 13539086

[3] Ruetten S, Komp M, Merk H, Godolias G. Full-endoscopic anterior decompression versus conventional anterior decompression and fusion in cervical disc herniations. Int Orthop. 2009;33(6):1677–82. doi: 10.1007/s00264-008-0684-y 19015851 PMC2899164

[4] Yang J-S, Chu L, Chen L, Chen F, Ke Z-Y, Deng Z-L. Anterior or posterior approach of full-endoscopic cervical discectomy for cervical intervertebral disc herniation? A comparative cohort study. Spine (Phila Pa 1976). 2014;39(21):1743–50. doi: 10.1097/BRS.0000000000000508 25010095

[5] Kim K-T, Cho D-C, Sung J-K, Kim Y-B, Kim DH. Comparative Analysis between Total Disc Replacement and Posterior Foraminotomy for Posterolateral Soft Disc Herniation with Unilateral Radiculopathy: Clinical and Biomechanical Results of a Minimum 5 Years Follow-up. J Korean Neurosurg Soc. 2017;60(1):30–9. doi: 10.3340/jkns.2015.0506.004 28061490 PMC5223749

[6] Epstein NE. A Review of Complication Rates for Anterior Cervical Diskectomy and Fusion (ACDF). Surg Neurol Int. 2019;10:100. doi: 10.25259/SNI-191-2019 31528438 PMC6744804

[7] Xiaobing Z, Xingchen L, Honggang Z, Xiaoqiang C, Qidong Y, Haijun M, et al. “U” route transforaminal percutaneous endoscopic thoracic discectomy as a new treatment for thoracic spinal stenosis. Int Orthop. 2019;43(4):825–32. doi: 10.1007/s00264-018-4145-y 30218183

[8] Ahn Y, Lee S-H, Shin S-W. Percutaneous endoscopic cervical discectomy: clinical outcome and radiographic changes. Photomed Laser Surg. 2005;23(4):362–8. doi: 10.1089/pho.2005.23.362 16144477

[9] Bohlman HH, Emery SE, Goodfellow DB, Jones PK. Robinson anterior cervical discectomy and arthrodesis for cervical radiculopathy. Long-term follow-up of one hundred and twenty-two patients. J Bone Joint Surg Am. 1993;75(9):1298–307. doi: 10.2106/00004623-199309000-00005 8408151

[10] Ruetten S, Komp M, Merk H, Godolias G. A new full-endoscopic technique for cervical posterior foraminotomy in the treatment of lateral disc herniations using 6.9-mm endoscopes: prospective 2-year results of 87 patients. Minim Invasive Neurosurg. 2007;50(4):219–26. doi: 10.1055/s-2007-985860 17948181

[11] Sahai N, Changoor S, Dunn CJ, Sinha K, Hwang KS, Faloon M, et al. Minimally Invasive Posterior Cervical Foraminotomy as an Alternative to Anterior Cervical Discectomy and Fusion for Unilateral Cervical Radiculopathy: A Systematic Review and Meta-analysis. Spine (Phila Pa 1976). 2019;44(24):1731–9. doi: 10.1097/BRS.0000000000003156 31343619

[12] Fang W, Huang L, Feng F, Yang B, He L, Du G, et al. Anterior cervical discectomy and fusion versus posterior cervical foraminotomy for the treatment of single-level unilateral cervical radiculopathy: a meta-analysis. J Orthop Surg Res. 2020;15(1):202. doi: 10.1186/s13018-020-01723-5 32487109 PMC7268305

[13] Tanaka N, Fujimoto Y, An HS, Ikuta Y, Yasuda M. The anatomic relation among the nerve roots, intervertebral foramina, and intervertebral discs of the cervical spine. Spine. 2000;25(3):286–91. doi: 10.1097/00007632-200002010-00005 10703098

[14] Joh J-Y, Choi G, Kong B-J, Park HS, Lee S-H, Chang SH. Comparative study of neck pain in relation to increase of cervical epidural pressure during percutaneous endoscopic lumbar discectomy. Spine (Phila Pa 1976). 2009;34(19):2033–8. doi: 10.1097/BRS.0b013e3181b20250 19675511

[15] Barakat M, Hussein Y. Anatomical study of the cervical nerve roots for posterior foraminotomy: cadaveric study. Eur Spine J. 2012;21(7):1383–8. doi: 10.1007/s00586-012-2158-6 22270247 PMC3389111

[16] Shishui L, Shangjun G, Feng L, Cheng L, Kefeng L, Shiguo Z, Yubin Z. Neurophysiological monitoring in treatment of lumbar disc herniation with percutaneous spinal endoscopy under general anesthesia. Chin J Neuromed. 2020;19(10):1035–1039. doi: 10.3760/cma.j.cn115354-20200528-00418

