# Supplementary material for: Efficacy evaluation of the cervical posterior “keyhole technique” in the treatment of cervical radiculopathy: A retrospective case series
Source: PLoS One. 2026 Oct 27;21(10):e0359633. doi: 10.1371/journal.pone.0359633 (PMC13712521; doi:10.1371/journal.pone.0359633)
Supplement: Supplementary file 2 [file pone.0359633.s003.docx]

**Manuscript ID:** PONE-D-26-28826R1
**Title:** Efficacy evaluation of the cervical posterior “keyhole technique” in the treatment of cervical radiculopathy: A retrospective case series

Dear Dr. Dean Chou and the Reviewers,

Thank you for your positive feedback and for reviewing our revised manuscript. We are thrilled to hear that both reviewers are satisfied with our previous revisions. We have carefully addressed the Academic Editor’s additional request regarding Figure 2, as well as the Journal Requirements regarding the reference list. Please find our point-by-point responses below.

**Reply to Journal Requirements:**

**Journal Requirement:** *Please review your reference list to ensure that it is complete and correct. If you have cited papers that have been retracted, please include the rationale for doing so in the manuscript text…*

**Response:**
We have carefully reviewed and verified our entire reference list item by item. We confirm that all cited references are highly relevant to this study, and no irrelevant literature has been cited. Furthermore, we have strictly checked our bibliography against current databases and can confirm that none of the cited papers have been retracted. The reference details have been thoroughly updated and completed to ensure full compliance with the journal’s formatting guidelines.

**Reply to the Academic Editor:**

**Editor’s Comment:** *I would like to review figure 2 which they made substantial revisions to, to provide a step by step explanation of the steps of the surgical procedure.*

**Response:**
Thank you for this constructive suggestion. We fully agree that a clear, step-by-step explanation significantly improves the readability and practical value of our manuscript.

In this revision, we have updated the figure legend for Figure 2 and the corresponding text in the manuscript to provide a detailed, step-by-step explanation of the posterior cervical “keyhole technique” under endoscopy.

Specifically, the step-by-step explanation for the intraoperative procedures (Figure 2B1-B4) has been added as follows:

- **Step 1 (Fig. 2B1):** After confirming the surgical level via fluoroscopy, the working channel and endoscope are sequentially inserted. Under endoscopic visualization, the soft tissues on the surface of the lamina and lateral mass joint are cleared. The “V-point” (indicated by a star) is exposed laterally through the interlaminar space, and a high-speed burr is used to drill the bone at the V-point.
- **Step 2 (Fig. 2B2):** A Kerrison rongeur is used to bite off the residual thin layer of bone, further exposing the V-point.
- **Step 3 (Fig. 2B3):** The lateral edge of the ligamentum flavum and the dorsal membranous structure of the nerve root are exposed. After identifying the nerve root, a partial resection of the ligamentum flavum is performed to further decompress the dorsal aspect of the nerve root canal.
- **Step 4 (Fig. 2B4):** A nerve hook is used to gently retract the nerve root. The axilla and shoulder regions of the nerve root are carefully explored, and the herniated disc material (nucleus pulposus) is removed to achieve complete release and mobilization of the nerve root.

We have incorporated these steps into the revised manuscript (highlighted in Track Changes). We hope this detailed explanation meets your requirements and perfectly illustrates the surgical procedure.

**Reply to Reviewer #2:**

**Reviewer #2’s Comment:** *The authors have appropriately addressed all of the concerns raised in the prior review. The revisions have improved the accuracy and clarity of the manuscript, and no further comments are warranted.*

**Response:**
Thank you very much for your time, expertise, and positive evaluation of our manuscript. Your constructive comments during the previous round of review have greatly improved the quality of our paper.

**Reply to Reviewer #3:**

**Reviewer #3’s Comment:** *Overall, I believe the revisions satisfactorily address the prior concerns, and I have no further substantive comments.*

**Response:**
We sincerely appreciate your thorough review and encouraging comments. We are glad that our revisions have met your expectations. Thank you for your valuable contribution to improving our study.
