# Supplementary material for: Efficacy evaluation of the cervical posterior “keyhole technique” in the treatment of cervical radiculopathy: A retrospective case series
Source: PLoS One. 2026 Oct 27;21(10):e0359633. doi: 10.1371/journal.pone.0359633 (PMC13712521; doi:10.1371/journal.pone.0359633)
Supplement: Supplementary file 3 [file pone.0359633.s002.docx]

**Dear Dr. Dean Chou,**
**Academic Editor, PLOS ONE**

Thank you and the reviewers for the thorough and constructive evaluation of our manuscript titled “[Efficacy evaluation of the cervical posterior “keyhole technique” in the treatment of cervical radiculopathy: A retrospective case series]”. We highly appreciate the time and effort you have dedicated to providing insightful feedback.

We have carefully revised the manuscript according to all comments. Below, we provide a point-by-point response to the Editor and the Reviewers. Changes in the revised manuscript are highlighted in the “Revised Manuscript” file.

**Part I: Response to Journal Requirements (Editor’s Comments)**

**1. Style Requirements:**
**Response:** We have reviewed PLOS ONE’s style templates and updated our manuscript’s formatting, including file naming, heading styles, and paragraph formatting, to ensure full compliance.

**2, 3, & 4. Funding Information, Discrepancies, and Role of Funder:**
**Response:** We apologize for the incorrect placement and discrepancies. We have completely removed all funding-related text from the Acknowledgments section. In the Cover Letter, we have provided the updated and matching grant numbers for the Funding Statement.

**5. Data Availability:**
**Response:** We confirm that all de-identified minimal underlying datasets necessary to replicate our study findings will be made freely accessible upon acceptance.

**6. ORCID iD:**
**Response:** The corresponding author’s ORCID iD has been successfully validated in the Editorial Manager system.

**7 & 8. Author Name Correction (Ruohui Tang):**
**Response:** We apologize for the typographical error. We have amended the author’s name to “Ruohui Tang” in both the Editorial Manager system and the manuscript file.

**9. Ethics Statement Location:**
**Response:** The ethics statement has been exclusively moved to the Methods section. Any mention of it in other sections has been deleted.

**10. Copyrighted Images (Figure 2):**
**Response:** All intraoperative images presented in this figure are original photographs taken personally by the authors during the surgical procedures.

**11. Tables:**
**Response:** All tables have been inserted directly into the main manuscript text immediately following the paragraph in which they are first cited, and individual table files have been removed.

**Part II: Response to Reviewer #1**

**General Comment:** *This study describes a technical note… The strengths of the article lie in the figures… There are, however, a few areas that the article should be improved in prior to publication.*
**Response:** We sincerely thank the reviewer for recognizing the clinical value of our study and the quality of our figures. Your constructive suggestions have significantly improved the depth and standalone value of our manuscript.

**Comment 1 (Introduction):** *The introduction should go into more detail involving the background of the field… Some combination of the following studies (Yang JS, Kim KT, Ruetten S) along with a description of their findings should be used in the introduction to highlight the divide in the field between posterior cervical foraminotomy and acdf…*
**Response:** We have expanded the Introduction to better reflect the historical divide and ongoing debate between ACDF and posterior cervical foraminotomy (PCF). We have incorporated the excellent studies you suggested (Yang JS, Kim KT, and Ruetten S) to highlight the biomechanical and clinical differences, thereby better establishing the background for our research. (Page [3], Lines [88-93]).

**Comment 2 (Methods - Steroids):** *The use of steroids can be a major confounding factor… Can the authors comment to whether any topical steroids were used? Also did all patients receive a dose of steroids for post operative nausea/vomiting or was this on a case by case basis?*
**Response:**  Thank you for raising this important point. We did not use local steroids during the procedure. Regarding systemic steroids, they were not used routinely. Specifically, only three patients who experienced limb numbness within 24 hours postoperatively received an intravenous administration of 60 mg methylprednisolone to relieve nerve root edema. We have added this detail to the Methods and Results sections to ensure complete transparency. (Page [5], Lines [180-183]).

**Comment 3 (Quantitative Evaluation):** *It would be important for the readers to know whether the indications for the decompression were the same in both groups, and would be helpful if a figure with a representative MRI from each group was shown to help guide the reader/surgeon in the decision making process outlined.*
**Response:** We appreciate this insightful comment. In our practice, the decision to perform a discectomy is based on preoperative MRI findings combined with intraoperative exploration. If the posterior annulus fibrosus remains intact and the nerve root tension decreases significantly after microscopic bony decompression, the disc is not excised. We believe that precise posterior bony and ligamentous decompression is the key to alleviating nerve root compression symptoms. We have detailed these surgical indications in the Methods section.

**Comment 4 (Figures):** *Ideally a figure in between figure 2 and 3 a figure can be added that can demonstrate the different technical steps of the procedure…*
**Response:** We strongly agree that a step-by-step intraoperative figure increases the reliability and standardization of the procedure. Accordingly, we have updated **Figure 2** by incorporating a series of intraoperative endoscopic images, including the identification of the V-point, utilizing the burr for foraminotomy, removing the ligamentum flavum, and exposing the nerve root.

**Part III: Response to Reviewer #2**

**General Comment:** *The clinical technique and core message are of interest, but the manuscript requires revisions.*
**Response:** We thank the reviewer for the positive assessment of our core message and for the meticulous review. We have addressed all your concerns below.

**Comment 1:** *The manuscript states that cervical radiculopathy falls under “degenerative cervical myelopathy (DCM)” terminology. This should be corrected or removed.*
**Response:** We apologize for the inaccurate terminology. We have removed the term “degenerative cervical myelopathy (DCM)” and corrected the text to specifically focus on “cervical spondylotic radiculopathy (CSR)” throughout the manuscript to avoid confusion. (Page [2], Lines [80-83]).

**Comment 2:** *“volume of excised nucleus pulposus” is stated to show no correlation with outcome, but no methodology for measuring or analyzing this variable appears anywhere…*
**Response:** We apologize for this expression error. We did not accurately measure the exact volume of the excised nucleus pulposus. Our intended meaning was to compare clinical outcomes based on “whether discectomy was performed or not.” We have corrected this phrasing throughout the manuscript. (Page [2], Lines [65-67]).

**Comment 3:** *Inconsistent study period across sections. Please address.*
**Response:** We apologize for the typo. We have carefully checked and unified the study period dates across the Abstract, Methods, and Results sections to ensure absolute consistency.

**Comment 4:** *Abstract/Methods state decompression parameters were quantified via “MRI/CT,” but Results only describe CT-based measurements in the imaging subgroup. Please clarify or correct.*
**Response:** We apologize for the inaccurate expression. We only quantified the parameters of bony decompression, which was strictly performed using postoperative CT scans. The mention of MRI for quantitative morphological measurement was an error, and we have corrected this in the Abstract and Methods.

**Comment 5:** *Recurrence (n=2) discussed but not reported as a Result.*
**Response:** Thank you for pointing out this omission. We have now explicitly reported in the Results section that two patients experienced a recurrence of symptoms and subsequently underwent Anterior Cervical Discectomy and Fusion (ACDF) surgery. (Page [5], Lines [211-213]).

**Part IV: Response to Reviewer #3**

**General Comment:** *The topic is clinically relevant, but the manuscript requires major revision because the rationale, patient selection, statistical reporting, and conclusions are not sufficiently clear or well supported.*
**Response:** We express our sincere gratitude for your rigorous and highly professional critique. Your comments have prompted us to significantly improve the methodology reporting, statistical presentation, and the objective tone of our conclusions.

**Comment 1 (Introduction):** *Prevalence of CSR (60%) requires a citation. Refocus background on radiculopathy, not ACDF/myelopathy. Clarify the specific knowledge gap and objective.*
**Response:** Following your advice, we have extensively rewritten the Introduction. We agree that the introduction was too broad. We have completely restructured the Introduction to focus strictly on cervical radiculopathy, posterior cervical foraminotomy, and endoscopic approaches. We have removed the distracting discussions regarding myelopathy. Furthermore, we clearly stated our primary objective at the end of the introduction: to evaluate the clinical efficacy and safety of this technique, and specifically to investigate whether the extent of bony decompression or the addition of discectomy provides further clinical benefits. (Page [2], Lines [80-83, 95-105]).

**Comment 2 (Methods - Patient Selection):** *Formal inclusion and exclusion criteria are needed…*
**Response:** We have added a dedicated sub-section in the Methods titled “Patient Selection,” clearly outlining the formal Inclusion criteria (e.g., single-level unilateral CSR, failure of conservative treatment > 6 weeks) and Exclusion criteria (e.g., cervical instability, myelopathy, severe calcified disc, previous cervical surgery). (Page [3], Lines [118-125]).

**Comment 3 (Methods - Radiology):** *Clarify who performed the radiological decompression measurements… Were assessors blinded?*
**Response:** We have clarified in the Methods section that all radiological measurements were performed independently by two spine surgeons who were not involved in the surgical procedures and were strictly blinded to the patients’ clinical outcomes. (Page [5], Lines [191-194]).

**Comment 4 (Results - Hospital Stay):** *The average hospital stay of 6.2 days appears high… clarify whether this reflects local practice, insurance…*
**Response:** Thank you for allowing us to clarify this. The relatively long hospital stay was not due to postoperative complications or delayed recovery. Instead, it was strictly due to the time required for routine preoperative examinations and scheduling delays at our local hospital (for example, scheduling an MRI scan typically requires a 1-to-3 day waiting period as an inpatient). We have added a brief explanation in the Discussion section to contextualize this finding for international readers. (Page [6-7], Lines [261-268]).

**Comment 5 (Results - Organization):** *Move methodological details to methods. Interpretive statements to discussion.*
**Response:** We have thoroughly reorganized the Results section. All descriptions of how CT measurements were taken have been moved to the Methods section, and subjective interpretations have been relocated to the Discussion section.

**Comment 6 (Results - Subgroups):** *Specify what criteria determined whether a patient underwent foraminotomy alone versus foraminotomy with discectomy.*
**Response:** In our practice, the decision to perform a discectomy is based on preoperative MRI findings combined with intraoperative exploration. If the posterior annulus fibrosus remains intact and the nerve root tension decreases significantly after microscopic bony decompression, the disc is not excised. We believe that precise posterior bony and ligamentous decompression is the key to alleviating nerve root compression symptoms. We have detailed these surgical indications in the Methods section. (Page [4], Lines [163-169]).

**Comment 7 (Results - Table 1 P-values):** *Table 1 should provide exact p values rather than reporting only P < 0.05.*
**Response:** We have updated Table 1 (and all text) to report exact P-values to allow readers to better judge the magnitude of the improvements.

**Comment 8 (Results - Only 20 post-op CTs):** *Explain why only 20 patients underwent postoperative CT assessment and whether these patients differed from the remaining cohort.*
**Response:** This is a crucial point. The lack of postoperative CT for the remaining 20 patients was deeply rooted in our local clinical reality. Primarily, due to rapid postoperative discharge (often on postoperative day 1 or 2), the window to schedule scans was narrow. More importantly, under the strict health insurance payment policies (DRG/DIP systems in China), routine postoperative CTs are often not fully covered. This additional financial burden led to direct patient refusal, particularly when their radiating pain had already significantly relieved immediately after surgery. There were no demographic or baseline clinical differences between the 20 patients who received scans and the 20 who did not. We have explicitly added this explanation to the Results. (Page [6], Lines [223-227]).

**Comment 9 (Results - Missing Data):** *State how many patients had complete clinical follow-up, imaging follow-up…*
**Response:** Thank you for this insightful observation. We chose 6 months postoperatively as the standard endpoint for our primary statistical analysis because the clinical data for all 40 included patients was most complete and comprehensive at this specific time point. In our real-world clinical practice, although some patients have been followed up for more than a year, others did not return for outpatient evaluations after 6 months (often due to satisfactory symptom relief and the inconvenience of traveling back to the hospital). To ensure the consistency and reliability of our statistical analysis, we strictly utilized the complete 6-month dataset. However, we completely agree that the lack of uniform long-term follow-up data is a shortcoming. We have now added this point as a limitation in the Discussion section. (Page [8], Lines [316-319]).

**Comment 10 (Results - Underpowered Subgroup):** *With 25 and 15 patients… lack of statistical significance does not establish equivalence.*
**Response:** We completely agree with your insightful critique. We acknowledge that the subgroup comparison (n=25 vs. n=15) is significantly underpowered. We have revised the Discussion section to explicitly state that the lack of statistical significance does *not* establish clinical equivalence between the two approaches, and we have reframed our discussion as a preliminary clinical observation rather than a definitive conclusion. (Page [7], Lines [272-277]).

**Comment 11, 12, 13, 14 (Conclusions):** *Conclusions are too strong… mechanistic claim not supported… avoid ACDF conclusions… short follow-up.*
**Response:** Thank you for pointing this out. We have carefully reviewed the manuscript and toned down our conclusions throughout. We have removed any comparative claims suggesting superiority or equivalence to ACDF or open posterior foraminotomy. ACDF is now only mentioned strictly as background context. We also explicitly stated in the Limitations section that the lack of a control group prevents us from making any direct comparisons regarding efficacy and safety.

We agree that our study design and statistical power are insufficient to make this mechanistic claim. We have modified the Discussion section to soften this point. We now state that while our findings align with the concept of targeted decompression, the study does not have sufficient strength to definitively claim that clinical efficacy relies solely on targeted neural decompression rather than wider laminoforaminotomy.

We fully acknowledge that 6 months is a short follow-up period, which limits our ability to evaluate long-term outcomes. We have explicitly added this to the Limitations section, specifically highlighting that a 6-month follow-up is insufficient for assessing late recurrences, delayed spinal instability, delayed complications, and the long-term durability of symptom relief.

We believe that the manuscript has been substantially improved thanks to the reviewers’ comments. We hope that the revised manuscript now meets the high standards for publication in PLOS ONE.

Thank you again for your time and consideration.

Sincerely,

Zhaowen Zhou
