# Supplementary material for: Efficacy evaluation of the cervical posterior “keyhole technique” in the treatment of cervical radiculopathy: A retrospective case series
Source: PLoS One. 2026 Oct 27;21(10):e0359633. doi: 10.1371/journal.pone.0359633 (PMC13712521; doi:10.1371/journal.pone.0359633)
Supplement: Peer review history

# Peer review: Decision Letter 0

**DOI:** 10.1371/journal.pone.0359633.r001

**Authors:** Dean Chou

7 Jul 2026

Dear Dr. Zhou,

Thank you for submitting your manuscript to PLOS ONE. After careful consideration, we feel that it has merit but does not fully meet PLOS ONE’s publication criteria as it currently stands. Therefore, we invite you to submit a revised version of the manuscript that addresses the points raised during the review process.

==============================

**I would be happy to entertain a revision if you can address the reviewers' comments adequately.**

==============================

Please submit your revised manuscript by Sep 05 2026 11:59PM. If you will need more time than this to complete your revisions, please reply to this message or contact the journal office at plosone@plos.org. When you're ready to submit your revision, log on to https://www.editorialmanager.com/pone/ and select the 'Submissions Needing Revision' folder to locate your manuscript file.

- A letter that responds to each point raised by the academic editor and reviewer(s). You should upload this letter as a separate file labeled 'Response to Reviewers'.
- A marked-up copy of your manuscript that highlights changes made to the original version. You should upload this as a separate file labeled 'Revised Manuscript with Track Changes'.
- An unmarked version of your revised paper without tracked changes. You should upload this as a separate file labeled 'Manuscript'.

If you would like to make changes to your financial disclosure, please include your updated statement in your cover letter. Guidelines for resubmitting your figure files are available below the reviewer comments at the end of this letter.

If applicable, we recommend that you deposit your laboratory protocols in protocols.io to enhance the reproducibility of your results. Protocols.io assigns your protocol its own identifier (DOI) so that it can be cited independently in the future. For instructions see: https://journals.plos.org/plosone/s/submission-guidelines#loc-laboratory-protocols. Additionally, PLOS ONE offers an option for publishing peer-reviewed Lab Protocol articles, which describe protocols hosted on protocols.io. Read more information on sharing protocols at https://plos.org/protocols?utm_medium=editorial-email&utm_source=authorletters&utm_campaign=protocols.

As the corresponding author, your ORCID iD is verified in the submission system and will appear in the published article. PLOS supports the use of ORCID, and we encourage all coauthors to register for an ORCID iD and use it as well. Please encourage your coauthors to verify their ORCID iD within the submission system before final acceptance, as unverified ORCID iDs will not appear in the published article. *Only*  the individual author can complete the verification step; PLOS staff *cannot*  verify ORCID iDs on behalf of authors.

We look forward to receiving your revised manuscript.

Kind regards,

Dean Chou, MD

Academic Editor

PLOS One

Journal Requirements:

When submitting your revision, we need you to address these additional requirements.

1. Please ensure that your manuscript meets PLOS ONE's style requirements, including those for file naming. The PLOS ONE style templates can be found at

https://journals.plos.org/plosone/s/file?id=wjVg/PLOSOne_formatting_sample_main_body.pdf and

https://journals.plos.org/plosone/s/file?id=ba62/PLOSOne_formatting_sample_title_authors_affiliations.pdf

2. Thank you for stating the following in the Acknowledgments Section of your manuscript:

“The study was supported by Yunnan Fundamental Research Projects, No. 202401AY070001-158 and Kunming Health Science and Technology Talent Cultivation Project, 2025-SW-040.”

We note that you have provided funding information that is not currently declared in your Funding Statement. However, funding information should not appear in the Acknowledgments section or other areas of your manuscript. We will only publish funding information present in the Funding Statement section of the online submission form.

Please remove any funding-related text from the manuscript and let us know how you would like to update your Funding Statement. Currently, your Funding Statement reads as follows:

“The study was supported by Yunnan Fundamental Research Projects, No. 202401AY070001-158 and Kunming Health Science and Technology Talent Cultivation Project, 2025-SW-040.”

Please include your amended statements within your cover letter; we will change the online submission form on your behalf.

3. We note that the grant information you provided in the ‘Funding Information’ and ‘Financial Disclosure’ sections do not match.

When you resubmit, please ensure that you provide the correct grant numbers for the awards you received for your study in the ‘Funding Information’ section.

4. Thank you for stating the following financial disclosure:

“The study was supported by Yunnan Fundamental Research Projects, No. 202401AY070001-158 and Kunming Health Science and Technology Talent Cultivation Project, 2025-SW-040.”

Please state what role the funders took in the study.  If the funders had no role, please state: "The funders had no role in study design, data collection and analysis, decision to publish, or preparation of the manuscript."

If this statement is not correct you must amend it as needed.

Please include this amended Role of Funder statement in your cover letter; we will change the online submission form on your behalf.

5. When completing the data availability statement of the submission form, you indicated that you will make your data available on acceptance. We strongly recommend all authors decide on a data sharing plan before acceptance, as the process can be lengthy and hold up publication timelines. Please note that, though access restrictions are acceptable now, your entire data will need to be made freely accessible if your manuscript is accepted for publication. This policy applies to all data except where public deposition would breach compliance with the protocol approved by your research ethics board. If you are unable to adhere to our open data policy, please kindly revise your statement to explain your reasoning and we will seek the editor's input on an exemption. Please be assured that, once you have provided your new statement, the assessment of your exemption will not hold up the peer review process.

6. PLOS requires an ORCID iD for the corresponding author in Editorial Manager on papers submitted after December 6th, 2016. Please ensure that you have an ORCID iD and that it is validated in Editorial Manager. To do this, go to ‘Update my Information’ (in the upper left-hand corner of the main menu), and click on the Fetch/Validate link next to the ORCID field. This will take you to the ORCID site and allow you to create a new iD or authenticate a pre-existing iD in Editorial Manager.

7. Please amend the manuscript submission data (via Edit Submission) to include author “Ruohui Tang”.

8. Please amend your authorship list in your manuscript file to include author “Tang Rruohui”.

9. Your ethics statement should only appear in the Methods section of your manuscript. If your ethics statement is written in any section besides the Methods, please delete it from any other section.

10. We note that Figure 2 in your submission contain copyrighted images. All PLOS content is published under the Creative Commons Attribution License (CC BY 4.0), which means that the manuscript, images, and Supporting Information files will be freely available online, and any third party is permitted to access, download, copy, distribute, and use these materials in any way, even commercially, with proper attribution. For more information, see our copyright guidelines: http://journals.plos.org/plosone/s/licenses-and-copyright.

We require you to either (1) present written permission from the copyright holder to publish these figures specifically under the CC BY 4.0 license, or (2) remove the figures from your submission:

1. You may seek permission from the original copyright holder of Figure(s) [#] to publish the content specifically under the CC BY 4.0 license.

We recommend that you contact the original copyright holder with the Content Permission Form (http://journals.plos.org/plosone/s/file?id=7c09/content-permission-form.pdf) and the following text:

“I request permission for the open-access journal PLOS ONE to publish XXX under the Creative Commons Attribution License (CCAL) CC BY 4.0 (http://creativecommons.org/licenses/by/4.0/). Please be aware that this license allows unrestricted use and distribution, even commercially, by third parties. Please reply and provide explicit written permission to publish XXX under a CC BY license and complete the attached form.”

Please upload the completed Content Permission Form or other proof of granted permissions as an "Other" file with your submission.

In the figure caption of the copyrighted figure, please include the following text: “Reprinted from [ref] under a CC BY license, with permission from [name of publisher], original copyright [original copyright year].”

2. If you are unable to obtain permission from the original copyright holder to publish these figures under the CC BY 4.0 license or if the copyright holder’s requirements are incompatible with the CC BY 4.0 license, please either i) remove the figure or ii) supply a replacement figure that complies with the CC BY 4.0 license. Please check copyright information on all replacement figures and update the figure caption with source information. If applicable, please specify in the figure caption text when a figure is similar but not identical to the original image and is therefore for illustrative purposes only.

11. Please include your tables as part of your main manuscript and remove the individual files. Please note that supplementary tables (should remain/ be uploaded) as separate "supporting information" files

12. If the reviewer comments include a recommendation to cite specific previously published works, please review and evaluate these publications to determine whether they are relevant and should be cited. There is no requirement to cite these works unless the editor has indicated otherwise.

[Note: HTML markup is below. Please do not edit.]

Reviewers' comments:

Reviewer's Responses to Questions

**Comments to the Author**

1. Is the manuscript technically sound, and do the data support the conclusions?

Reviewer #1: Yes

Reviewer #2: Yes

Reviewer #3: Partly

**********

2. Has the statistical analysis been performed appropriately and rigorously?

Reviewer #1: Yes

Reviewer #2: No

Reviewer #3: Yes

**********

3. Have the authors made all data underlying the findings in their manuscript fully available?

The PLOS Data policy

Reviewer #1: Yes

Reviewer #2: Yes

Reviewer #3: Yes

**********

4. Is the manuscript presented in an intelligible fashion and written in standard English?

Reviewer #1: Yes

Reviewer #2: Yes

Reviewer #3: Yes

**********

Reviewer #1: This study describes a technical note on a retrospective case series of endoscopic posterior keyhole decompression/foraminotomy +/- medial facetectomy. This is an area of ongoing active research and this article adds to the body of literature demonstrating that posterior endoscopic cervical foraminotomy is a safe technique. The strengths of the article lie in the figures which show an excellent demonstration of the posterior keyhole bony removal, along with the subgroup analysis comparing discectomy versus no-discectomy patients. This area particularly may help guide future clinical care as it shows minimal additional benefit with discectomy compared to foraminotomy alone. There are, however, a few areas that the article should be improved in prior to publication.

Introduction:

- The introduction should go into more detail involving the background of the field. In the discussion the authors site the Ruetten study along with 2 others but there are many such studies that should be highlighted as this has been an increasing area of publication over the last 20 years. Some combination of the following studies along with a description of their findings should be used in the introduction to highlight the divide in the field between posterior cervical foraminotomy and acdf for treatment of radiculopathy. See below for a list of such studies. Not all need to be referenced individually, but citation of the field can help improve this publications standalone value.

1. Yang JS, Chu L, Chen L, Chen F, Ke ZY, Deng ZL. Anterior or posterior approach of full-endoscopic cervical discectomy for cervical intervertebral disc herniation? A comparative cohort study. Spine (Phila Pa 1976). 2014;39:1743–50.

2. Kim KT, Cho DC, Sung JK, Kim YB, Kim DH. Comparative analysis between total disc replacement and posterior foraminotomy for posterolateral soft disc herniation with unilateral radiculopathy: clinical and biomechanical results of a minimum 5 years follow-up. J Korean Neurosurg Soc. 2017;60:30–9.

3. Ruetten S, Komp M, Merk H, Godolias G. Full- endoscopic anterior decompression versus conventional anterior decompression and fusion in cervical disc herniations. Int Orthop. 2009;33:1677–82.

4. Park YK, Moon HJ, Kwon TH, Kim JH. Long-term outcomes following anterior foraminotomy for one- or two-level cervical radiculopathy. Eur Spine J. 2013;22:1489–96.

5. Kim KT, Kim YB. Comparison between open procedure and tubular retractor assisted procedure for cervical radiculopathy: results of a randomized controlled study. J Korean Med Sci. 2009;24:649–53.

6. Ruetten S, Komp M, Merk H, Godolias G. Full- endoscopic cervical posterior foraminotomy for the operation of lateral disc herniations using 5.9-mm endoscopes: a prospective, randomized, controlled study. Spine (Phila Pa 1976). 2008;33:940–8.

7. Won S, Kim CK, Chung CK, et al. Clinical outcomes of single-level posterior percutaneous endoscopic cervical foraminotomy for patients with less cervical lordosis. J Minim Invasive Spine Surg Tech. 2016;1:11–7.

8. Kim CH, Shin KH, Chung CK, Park SB, Kim JH. Changes in cervical sagittal alignment after single- level posterior percutaneous endoscopic cervical diskectomy. Global Spine J. 2015;5:31–8.

9. Kim CH, Kim KT, Chung CK, et al. Minimally invasive cervical foraminotomy and diskectomy for laterally located soft disk herniation. Eur Spine J. 2015;24:3005–12. 3

10. Kim CH, Chung CK, Kim HJ, Jahng TA, Kim DG. Early outcome of posterior cervical endoscopic discectomy: an alternative treatment choice for physically/socially active patients. J Korean Med Sci. 2009;24:302–6.

11. Ruetten S, Komp M, Merk H, Godolias G. A new full-endoscopic technique for cervical posterior foraminotomy in the treatment of lateral disc herniations using 6.9-mm endoscopes: prospective 2-year results of 87 patients. Minim Invasive Neurosurg. 2007;50:219–26.

Materials and Methods

- Pg. 4 Line 155-157 “Additionally, depending on the severity of residual symptoms, adjunct therapies including osmotic diuretics, low-dose glucocorticoids, or neurotrophic agents”

o The use of steroids can be a major confounding factor in the early outcomes for patients. Can the authors comment to whether any topical steroids were used? Also did all patients receive a dose of steroids for post operative nausea/vomiting or was this on a case by case basis?

Quantitative Evaluation

- Pg. 5, line 210-212 “Intergroup analysis revealed no statistically significant differences between these two subgroups regarding operative time, intraoperative blood loss, or hospitalization costs (all P > 0.05).”

o This is the most unique feature of this paper. Notably a few additional details should be provided regarding differences in baseline imaging when discectomy was pursued versus when it was not. It would be important for the readers to know whether the indications for the decompression were the same in both groups, and would be helpful if a figure with a representative MRI from each group was shown to help guide the reader/surgeon in the decision making process outlined.

Figures

- Ideally a figure in between figure 2 and 3 a figure can be added that can demonstrate the different technical steps of the procedure such as with Introducing the endoscope at V point, clearing of soft tissue with the bipolar/punch, Identifying the V point, Utilizing the diamond burr/drill for foraminotomy, Dissection/removal of ligamentum flavum. These steps have been published before and are not of critical importance but help increase the reliability of the findings as they standardize the surgical approach.

Reviewer #2: This retrospective case series (n=40) evaluates endoscopic posterior "keyhole" decompression for cervical radiculopathy and reports favorable short-term outcomes with no correlation between decompression extent and clinical improvement. The clinical technique and core message are of interest, but the manuscript requires revisions.

1. The manuscript states that cervical radiculopathy falls under "degenerative cervical myelopathy (DCM)" terminology. This should be corrected or removed.

2. "volume of excised nucleus pulposus" is stated to show no correlation with outcome, but no methodology for measuring or analyzing this variable appears anywhere in the Methods or Results.

3. Inconsistent study period across sections. Please address.

4. Abstract/Methods state decompression parameters were quantified via "MRI/CT," but Results only describe CT-based measurements in the imaging subgroup. Please clarify or correct.

5. Recurrence (n=2) discussed but not reported as a Result.

Reviewer #3: This retrospective case series evaluates endoscopic posterior “keyhole” decompression for single-level cervical radiculopathy and reports improvement in VAS, JOA, and NDI scores at discharge and 6 months. The topic is clinically relevant, but the manuscript requires major revision because the rationale, patient selection, statistical reporting, and conclusions are not sufficiently clear or well supported.

Introduction and background

• The statement regarding the prevalence of cervical spondylotic radiculopathy, reported as approximately 60% of cervical spondylosis cases, requires a citation.

• The introduction discusses myelopathy and ACDF, but the actual focus of the study is endoscopic posterior decompression for radiculopathy. The background should be reframed around cervical radiculopathy, posterior cervical foraminotomy, endoscopic approaches, and the specific knowledge gap this paper is trying to address.

• The authors should better explain why this study is needed. Is the main question safety, clinical efficacy, optimal decompression size, need for discectomy, or comparison with ACDF? At present, the objective is somewhat broad.

Methods

• The authors need to clarify how patients were selected for this retrospective series. It is not clear whether all eligible patients during the study period were included. Formal inclusion and exclusion criteria are needed. Although diagnostic criteria are described, the manuscript does not clearly state which patients were excluded and why.

• The manuscript should clarify who performed the radiological decompression measurements. Were these assessed by the treating surgeon, an independent spine surgeon, or a radiologist? Were assessors blinded to clinical outcomes?

Results

• The average hospital stay of 6.2 days appears high for patients undergoing endoscopic decompression for cervical radiculopathy. The authors should clarify whether this reflects local practice, insurance or health system factors, postoperative monitoring protocols, or patient comorbidity. This is important for interpreting the generalizability of the results.

• The results section needs improved organization. For example, the imaging findings section includes methodological details about how measurements were taken, which should be moved to the methods. It also includes interpretive statements that would be more appropriate for the discussion.

• The statement, “Based on preoperative pathology and surgical planning, the cohort was divided into two subgroups,” requires further explanation. The authors should specify what criteria determined whether a patient underwent foraminotomy alone versus foraminotomy with discectomy.

• Table 1 should provide exact p values rather than reporting only P < 0.05. Exact p values and confidence intervals would allow readers to better judge the magnitude and reliability of the observed improvements.

• Table 2 includes only 20 cases across C4/5, C5/6, and C6/7, whereas the full cohort includes 40 patients. The authors need to explain why only 20 patients underwent postoperative CT assessment and whether these patients differed from the remaining cohort.

• More information is needed regarding missing data and follow-up. The authors should state how many patients had complete clinical follow-up, how many had imaging follow-up, and whether any patients were excluded from specific analyses.

• The subgroup comparison between decompression alone and decompression plus nucleus pulposus removal is also underpowered. With 25 and 15 patients in each group, the lack of statistical significance does not establish equivalence between the two approaches.

Conclusions

• The conclusions are too strong for the evidence presented. This is a retrospective, single-center case series without a control group, so it cannot establish that this technique is superior or equivalent to ACDF or open posterior foraminotomy.

• The conclusion that clinical efficacy depends on targeted neural decompression rather than disc removal or wider laminoforaminotomy is not fully supported. The study does not have sufficient power or design strength to make this mechanistic claim.

• Because there is no comparison group, the manuscript should avoid drawing conclusions about ACDF beyond general background discussion.

• The follow-up period of 6 months is short, particularly for assessing recurrence, instability, delayed complications, and durability of symptom relief.

Overall, the paper addresses a relevant clinical topic, but it needs major revision with clearer framing, formal inclusion and exclusion criteria, better explanation of missing imaging data, improved statistical reporting, and more cautious conclusions.

**********

what does this mean?). If published, this will include your full peer review and any attached files.

If you choose “no”, your identity will remain anonymous but your review may still be made public.

**Do you want your identity to be public for this peer review?**  For information about this choice, including consent withdrawal, please see our Privacy Policy

Reviewer #1: **Yes:**  Pavan Upadhyayula

Reviewer #2: No

Reviewer #3: No

**********

[NOTE: If reviewer comments were submitted as an attachment file, they will be attached to this email and accessible via the submission site. Please log into your account, locate the manuscript record, and check for the action link "View Attachments". If this link does not appear, there are no attachment files.]

To ensure your figures meet our technical requirements, please review our figure guidelines: https://journals.plos.org/plosone/s/figures

You may also use PLOS’s free figure tool, NAAS, to help you prepare publication quality figures: https://journals.plos.org/plosone/s/figures#loc-tools-for-figure-preparation.

NAAS will assess whether your figures meet our technical requirements by comparing each figure against our figure specifications.

# Author comment: Author response to Decision Letter 1

**DOI:** 10.1371/journal.pone.0359633.r002

27 Aug 2026

Dear Dr. Dean Chou,

Academic Editor, PLOS ONE

Thank you and the reviewers for the thorough and constructive evaluation of our manuscript titled “[Efficacy evaluation of the cervical posterior “keyhole technique” in the treatment of cervical radiculopathy: A retrospective case series]”. We highly appreciate the time and effort you have dedicated to providing insightful feedback.

We have carefully revised the manuscript according to all comments. Below, we provide a point-by-point response to the Editor and the Reviewers. Changes in the revised manuscript are highlighted in the “Revised Manuscript” file.

________________________________________

Part I: Response to Journal Requirements (Editor’s Comments)

1. Style Requirements:

Response: We have reviewed PLOS ONE’s style templates and updated our manuscript’s formatting, including file naming, heading styles, and paragraph formatting, to ensure full compliance.

2, 3, & 4. Funding Information, Discrepancies, and Role of Funder:

Response: We apologize for the incorrect placement and discrepancies. We have completely removed all funding-related text from the Acknowledgments section. In the Cover Letter, we have provided the updated and matching grant numbers for the Funding Statement.

5. Data Availability:

Response: We confirm that all de-identified minimal underlying datasets necessary to replicate our study findings will be made freely accessible upon acceptance.

6. ORCID iD:

Response: The corresponding author’s ORCID iD has been successfully validated in the Editorial Manager system.

7 & 8. Author Name Correction (Ruohui Tang):

Response: We apologize for the typographical error. We have amended the author’s name to “Ruohui Tang” in both the Editorial Manager system and the manuscript file.

9. Ethics Statement Location:

Response: The ethics statement has been exclusively moved to the Methods section. Any mention of it in other sections has been deleted.

10. Copyrighted Images (Figure 2):

Response: All intraoperative images presented in this figure are original photographs taken personally by the authors during the surgical procedures.

11. Tables:

Response: All tables have been inserted directly into the main manuscript text immediately following the paragraph in which they are first cited, and individual table files have been removed.

________________________________________

Part II: Response to Reviewer #1

General Comment: This study describes a technical note… The strengths of the article lie in the figures… There are, however, a few areas that the article should be improved in prior to publication.

Response: We sincerely thank the reviewer for recognizing the clinical value of our study and the quality of our figures. Your constructive suggestions have significantly improved the depth and standalone value of our manuscript.

Comment 1 (Introduction): The introduction should go into more detail involving the background of the field… Some combination of the following studies (Yang JS, Kim KT, Ruetten S) along with a description of their findings should be used in the introduction to highlight the divide in the field between posterior cervical foraminotomy and acdf…

Response: We have expanded the Introduction to better reflect the historical divide and ongoing debate between ACDF and posterior cervical foraminotomy (PCF). We have incorporated the excellent studies you suggested (Yang JS, Kim KT, and Ruetten S) to highlight the biomechanical and clinical differences, thereby better establishing the background for our research. (Page [3], Lines [88-93]).

Comment 2 (Methods - Steroids): The use of steroids can be a major confounding factor… Can the authors comment to whether any topical steroids were used? Also did all patients receive a dose of steroids for post operative nausea/vomiting or was this on a case by case basis?

Response: Thank you for raising this important point. We did not use local steroids during the procedure. Regarding systemic steroids, they were not used routinely. Specifically, only three patients who experienced limb numbness within 24 hours postoperatively received an intravenous administration of 60 mg methylprednisolone to relieve nerve root edema. We have added this detail to the Methods and Results sections to ensure complete transparency. (Page [5], Lines [180-183]).

Comment 3 (Quantitative Evaluation): It would be important for the readers to know whether the indications for the decompression were the same in both groups, and would be helpful if a figure with a representative MRI from each group was shown to help guide the reader/surgeon in the decision making process outlined.

Response: We appreciate this insightful comment. In our practice, the decision to perform a discectomy is based on preoperative MRI findings combined with intraoperative exploration. If the posterior annulus fibrosus remains intact and the nerve root tension decreases significantly after microscopic bony decompression, the disc is not excised. We believe that precise posterior bony and ligamentous decompression is the key to alleviating nerve root compression symptoms. We have detailed these surgical indications in the Methods section.

Comment 4 (Figures): Ideally a figure in between figure 2 and 3 a figure can be added that can demonstrate the different technical steps of the procedure…

Response: We strongly agree that a step-by-step intraoperative figure increases the reliability and standardization of the procedure. Accordingly, we have updated Figure 2 by incorporating a series of intraoperative endoscopic images, including the identification of the V-point, utilizing the burr for foraminotomy, removing the ligamentum flavum, and exposing the nerve root.

________________________________________

Part III: Response to Reviewer #2

General Comment: The clinical technique and core message are of interest, but the manuscript requires revisions.

Response: We thank the reviewer for the positive assessment of our core message and for the meticulous review. We have addressed all your concerns below.

Comment 1: The manuscript states that cervical radiculopathy falls under “degenerative cervical myelopathy (DCM)” terminology. This should be corrected or removed.

Response: We apologize for the inaccurate terminology. We have removed the term “degenerative cervical myelopathy (DCM)” and corrected the text to specifically focus on “cervical spondylotic radiculopathy (CSR)” throughout the manuscript to avoid confusion. (Page [2], Lines [80-83]).

Comment 2: “volume of excised nucleus pulposus” is stated to show no correlation with outcome, but no methodology for measuring or analyzing this variable appears anywhere…

Response: We apologize for this expression error. We did not accurately measure the exact volume of the excised nucleus pulposus. Our intended meaning was to compare clinical outcomes based on “whether discectomy was performed or not.” We have corrected this phrasing throughout the manuscript. (Page [2], Lines [65-67]).

Comment 3: Inconsistent study period across sections. Please address.

Response: We apologize for the typo. We have carefully checked and unified the study period dates across the Abstract, Methods, and Results sections to ensure absolute consistency.

Comment 4: Abstract/Methods state decompression parameters were quantified via “MRI/CT,” but Results only describe CT-based measurements in the imaging subgroup. Please clarify or correct.

Response: We apologize for the inaccurate expression. We only quantified the parameters of bony decompression, which was strictly performed using postoperative CT scans. The mention of MRI for quantitative morphological measurement was an error, and we have corrected this in the Abstract and Methods.

Comment 5: Recurrence (n=2) discussed but not reported as a Result.

Response: Thank you for pointing out this omission. We have now explicitly reported in the Results section that two patients experienced a recurrence of symptoms and subsequently underwent Anterior Cervical Discectomy and Fusion (ACDF) surgery. (Page [5], Lines [211-213]).

________________________________________

Part IV: Response to Reviewer #3

General Comment: The topic is clinically relevant, but the manuscript requires major revision because the rationale, patient selection, statistical reporting, and conclusions are not sufficiently clear or well supported.

Response: We express our sincere gratitude for your rigorous and highly professional critique. Your comments have prompted us to significantly improve the methodology reporting, statistical presentation, and the objective tone of our conclusions.

Comment 1 (Introduction): Prevalence of CSR (60%) requires a citation. Refocus background on radiculopathy, not ACDF/myelopathy. Clarify the specific knowledge gap and objective.

Response: Following your advice, we have extensively rewritten the Introduction. We agree that the introduction was too broad. We have completely restructured the Introduction to focus strictly on cervical radiculopathy, posterior cervical foraminotomy, and endoscopic approaches. We have removed the distracting discussions regarding myelopathy. Furthermore, we clearly stated our primary objective at the end of the introduction: to evaluate the clinical efficacy and safety of this technique, and specifically to investigate whether the extent of bony decompression or the addition of discectomy provides further clinical benefits. (Page [2], Lines [80-83, 95-105]).

Comment 2 (Methods - Patient Selection): Formal inclusion and exclusion criteria are needed…

Response: We have added a dedicated sub-section in the Methods titled “Patient Selection,” clearly outlining the formal Inclusion criteria (e.g., single-level unilateral CSR, failure of conservative treatment > 6 weeks) and Exclusion criteria (e.g., cervical instability, myelopathy, severe calcified disc, previous cervical surgery). (Page [3], Lines [118-125]).

Comment 3 (Methods - Radiology): Clarify who performed the radiological decompression measurements… Were assessors blinded?

Response: We have clarified in the Methods section that all radiological measurements were performed independently by two spine surgeons who were not involved in the surgical procedures and were strictly blinded to the patients’ clinical outcomes. (Page [5], Lines [191-194]).

Comment 4 (Results - Hospital Stay): The average hospital stay of 6.2 days appears high… clarify whether this reflects local practice, insurance…

Response: Thank you for allowing us to clarify this. The relatively long hospital stay was not due to postoperative complications or delayed recovery. Instead, it was strictly due to the time required for routine preoperative examinations and scheduling delays at our local hospital (for example, scheduling an MRI scan typically requires a 1-to-3 day waiting period as an inpatient). We have added a brief explanation in the Discussion section to contextualize this finding for international readers. (Page [6-7], Lines [261-268]).

Comment 5 (Results - Organization): Move methodological details to methods. Interpretive statements to discussion.

Response: We have thoroughly reorganized the Results section. All descriptions of how CT measurements were taken have been moved to the Methods section, and subjective interpretations have been relocated to the Discussion section.

Comment 6 (Results - Subgroups): Specify what criteria determined whether a patient underwent foraminotomy alone versus foraminotomy with discectomy.

Response: In our practice, the decision to perform a discectomy is based on preoperative MRI findings combined with intraoperative exploration. If the posterior annulus fibrosus remains intact and the nerve root tension decreases significantly after microscopic bony decompression, the disc is not excised. We believe that precise posterior bony and ligamentous decompression is the key to alleviating nerve root compression symptoms. We have detailed these surgical indications in the Methods section. (Page [4], Lines [163-169]).

Comment 7 (Results - Table 1 P-values): Table 1 should provide exact p values rather than reporting only P < 0.05.

Response: We have updated Table 1 (and all text) to report exact P-values to allow readers to better judge the magnitude of the improvements.

Comment 8 (Results - Only 20 post-op CTs): Explain why only 20 patients underwent postoperative CT assessment and whether these patients differed from the remaining cohort.

Response: This is a crucial point. The lack of postoperative CT for the remaining 20 patients was deeply rooted in our local clinical reality. Primarily, due to rapid postoperative discharge (often on postoperative day 1 or 2), the window to schedule scans was narrow. More importantly, under the strict health insurance payment policies (DRG/DIP systems in China), routine postoperative CTs are often not fully covered. This additional financial burden led to direct patient refusal, particularly when their radiating pain had already significantly relieved immediately after surgery. There were no demographic or baseline clinical differences between the 20 patients who received scans and the 20 who did not. We have explicitly added this explanation to the Results. (Page [6], Lines [223-227]).

Comment 9 (Results - Missing Data): State how many patients had complete clinical follow-up, imaging follow-up…

Response: Thank you for this insightful observation. We chose 6 months postoperatively as the standard endpoint for our primary statistical analysis because the clinical data for all 40 included patients was most complete and comprehensive at this specific time point. In our real-world clinical practice, although some patients have been followed up for more than a year, others did not return for outpatient evaluations after 6 months (often due to satisfactory symptom relief and the inconvenience of traveling back to the hospital). To ensure the consistency and reliability of our statistical analysis, we strictly utilized the complete 6-month dataset. However, we completely agree that the lack of uniform long-term follow-up data is a shortcoming. We have now added this point as a limitation in the Discussion section. (Page [8], Lines [316-319]).

Comment 10 (Results - Underpowered Subgroup): With 25 and 15 patients… lack of statistical significance does not establish equivalence.

Response: We completely agree with your insightful critique. We acknowledge that the subgroup comparison (n=25 vs. n=15) is significantly underpowered. We have revised the Discussion section to explicitly state that the lack of statistical significance does not establish clinical equivalence between the two approaches, and we have reframed our discussion as a preliminary clinical observation rather than a definitive conclusion. (Page [7], Lines [272-277]).

Comment 11, 12, 13, 14 (Conclusions): Conclusions are too strong… mechanistic claim not supported… avoid ACDF conclusions… short follow-up.

Response: Thank you for pointing this out. We have carefully reviewed the manuscript and toned down our conclusions throughout. We have removed any comparative claims suggesting superiority or equivalence to ACDF or open posterior foraminotomy. ACDF is now only mentioned strictly as background context. We also explicitly stated in the Limitations section that the lack of a control group prevents us from making any direct comparisons regarding efficacy and safety.

We agree that our study design and statistical power are insufficient to make this mechanistic claim. We have modified the Discussion section to soften this point. We now state that while our findings align with the concept of targeted decompression, the study does not have sufficient strength to definitively claim that clinical efficacy relies solely on targeted neural decompression rather than wider laminoforaminotomy.

We fully acknowledge that 6 months is a short follow-up period, which limits our ability to evaluate long-term outcomes. We have explicitly added this to the Limitations section, specifically highlighting that a 6-month follow-up is insufficient for assessing late recurrences, delayed spinal instability, delayed complications, and the long-term durability of symptom relief.

________________________________________

We believe that the manuscript has been substantially improved thanks to the reviewers’ comments. We hope that the revised manuscript now meets the high standards for publication in PLOS ONE.

Thank you again for your time and consideration.

Sincerely,

Zhaowen Zhou

Attachment Submitted filename: Response.docx

# Peer review: Decision Letter 1

**DOI:** 10.1371/journal.pone.0359633.r003

**Authors:** Dean Chou

14 Sep 2026

Dear Dr. Zhou,

Thank you for submitting your manuscript to PLOS One. After careful consideration, we feel that it has merit but does not fully meet PLOS One’s publication criteria as it currently stands. Therefore, we invite you to submit a revised version of the manuscript that addresses the points raised during the review process.

Please submit your revised manuscript by Oct 14 2026 11:59PM. If you will need more time than this to complete your revisions, please reply to this message or contact the journal office at plosone@plos.org. When you're ready to submit your revision, log on to https://www.editorialmanager.com/pone/ and select the 'Submissions Needing Revision' folder to locate your manuscript file.

- A letter that responds to each point raised by the academic editor and reviewer(s). You should upload this letter as a separate file labeled 'Response to Reviewers'.
- A marked-up copy of your manuscript that highlights changes made to the original version. You should upload this as a separate file labeled 'Revised Manuscript with Track Changes'.
- An unmarked version of your revised paper without tracked changes. You should upload this as a separate file labeled 'Manuscript'.

If you would like to make changes to your financial disclosure, please include your updated statement in your cover letter. Guidelines for resubmitting your figure files are available below the reviewer comments at the end of this letter.

If applicable, we recommend that you deposit your laboratory protocols in protocols.io to enhance the reproducibility of your results. Protocols.io assigns your protocol its own identifier (DOI) so that it can be cited independently in the future. For instructions see: https://journals.plos.org/plosone/s/submission-guidelines#loc-laboratory-protocols. Additionally, PLOS One offers an option for publishing peer-reviewed Lab Protocol articles, which describe protocols hosted on protocols.io. Read more information on sharing protocols at https://plos.org/protocols?utm_medium=editorial-email&utm_source=authorletters&utm_campaign=protocols.

As the corresponding author, your ORCID iD is verified in the submission system and will appear in the published article. PLOS supports the use of ORCID, and we encourage all coauthors to register for an ORCID iD and use it as well. Please encourage your coauthors to verify their ORCID iD within the submission system before final acceptance, as unverified ORCID iDs will not appear in the published article. *Only*  the individual author can complete the verification step; PLOS staff *cannot*  verify ORCID iDs on behalf of authors.

We look forward to receiving your revised manuscript.

Kind regards,

Dean Chou, MD

Academic Editor

PLOS One

Journal Requirements:

If the reviewer comments include a recommendation to cite specific previously published works, please review and evaluate these publications to determine whether they are relevant and should be cited. There is no requirement to cite these works unless the editor has indicated otherwise.

Please review your reference list to ensure that it is complete and correct. If you have cited papers that have been retracted, please include the rationale for doing so in the manuscript text, or remove these references and replace them with relevant current references. Any changes to the reference list should be mentioned in the rebuttal letter that accompanies your revised manuscript. If you need to cite a retracted article, indicate the article’s retracted status in the References list and also include a citation and full reference for the retraction notice.

**Additional Editor Comments:**

I would like to review figure 2 which they made substantial revisions to, to provide a step by step explanation of the steps of the surgical procedure.

[Note: HTML markup is below. Please do not edit.]

Reviewers' comments:

Reviewer's Responses to Questions

**Comments to the Author**

Reviewer #2: All comments have been addressed

Reviewer #3: All comments have been addressed

**********

2. Is the manuscript technically sound, and do the data support the conclusions?

Reviewer #2: Yes

Reviewer #3: Yes

**********

3. Has the statistical analysis been performed appropriately and rigorously?

Reviewer #2: Yes

Reviewer #3: Yes

**********

4. Have the authors made all data underlying the findings in their manuscript fully available?

The PLOS Data policy

Reviewer #2: Yes

Reviewer #3: Yes

**********

5. Is the manuscript presented in an intelligible fashion and written in standard English?

Reviewer #2: Yes

Reviewer #3: Yes

**********

Reviewer #2: The authors have appropriately addressed all of the concerns raised in the prior review. The revisions have improved the accuracy and clarity of the manuscript, and no further comments are warranted.

Reviewer #3: The authors have adequately addressed the concerns raised in the previous review, and the manuscript has been substantially improved. The research question, inclusion/exclusion criteria, indications for foraminotomy versus discectomy, and radiological assessment have been appropriately clarified. The issue of incomplete postoperative imaging has also been explained and acknowledged as a limitation.

The Results and Discussion have been appropriately revised, particularly regarding the underpowered subgroup analysis and the avoidance of claims of equivalence or superiority. The study limitations are now adequately presented and appropriately contextualized.

Overall, I believe the revisions satisfactorily address the prior concerns, and I have no further substantive comments.

**********

what does this mean?). If published, this will include your full peer review and any attached files.

If you choose “no”, your identity will remain anonymous but your review may still be made public.

**Do you want your identity to be public for this peer review?**  For information about this choice, including consent withdrawal, please see our Privacy Policy

Reviewer #2: No

Reviewer #3: No

**********

[NOTE: If reviewer comments were submitted as an attachment file, they will be attached to this email and accessible via the submission site. Please log into your account, locate the manuscript record, and check for the action link "View Attachments". If this link does not appear, there are no attachment files.]

To ensure your figures meet our technical requirements, please review our figure guidelines: https://journals.plos.org/plosone/s/figures

You may also use PLOS’s free figure tool, NAAS, to help you prepare publication quality figures: https://journals.plos.org/plosone/s/figures#loc-tools-for-figure-preparation.

NAAS will assess whether your figures meet our technical requirements by comparing each figure against our figure specifications.

# Author comment: Author response to Decision Letter 2

**DOI:** 10.1371/journal.pone.0359633.r004

14 Sep 2026

Manuscript ID: PONE-D-26-28826R1

Title: Efficacy evaluation of the cervical posterior “keyhole technique” in the treatment of cervical radiculopathy: A retrospective case series

Dear Dr. Dean Chou and the Reviewers,

Thank you for your positive feedback and for reviewing our revised manuscript. We are thrilled to hear that both reviewers are satisfied with our previous revisions. We have carefully addressed the Academic Editor’s additional request regarding Figure 2, as well as the Journal Requirements regarding the reference list. Please find our point-by-point responses below.

________________________________________

Reply to Journal Requirements:

Journal Requirement: Please review your reference list to ensure that it is complete and correct. If you have cited papers that have been retracted, please include the rationale for doing so in the manuscript text…

Response:

We have carefully reviewed and verified our entire reference list item by item. We confirm that all cited references are highly relevant to this study, and no irrelevant literature has been cited. Furthermore, we have strictly checked our bibliography against current databases and can confirm that none of the cited papers have been retracted. The reference details have been thoroughly updated and completed to ensure full compliance with the journal’s formatting guidelines.

________________________________________

Reply to the Academic Editor:

Editor’s Comment: I would like to review figure 2 which they made substantial revisions to, to provide a step by step explanation of the steps of the surgical procedure.

Response:

Thank you for this constructive suggestion. We fully agree that a clear, step-by-step explanation significantly improves the readability and practical value of our manuscript.

In this revision, we have updated the figure legend for Figure 2 and the corresponding text in the manuscript to provide a detailed, step-by-step explanation of the posterior cervical “keyhole technique” under endoscopy.

Specifically, the step-by-step explanation for the intraoperative procedures (Figure 2B1-B4) has been added as follows:

• Step 1 (Fig. 2B1): After confirming the surgical level via fluoroscopy, the working channel and endoscope are sequentially inserted. Under endoscopic visualization, the soft tissues on the surface of the lamina and lateral mass joint are cleared. The “V-point” (indicated by a star) is exposed laterally through the interlaminar space, and a high-speed burr is used to drill the bone at the V-point.

• Step 2 (Fig. 2B2): A Kerrison rongeur is used to bite off the residual thin layer of bone, further exposing the V-point.

• Step 3 (Fig. 2B3): The lateral edge of the ligamentum flavum and the dorsal membranous structure of the nerve root are exposed. After identifying the nerve root, a partial resection of the ligamentum flavum is performed to further decompress the dorsal aspect of the nerve root canal.

• Step 4 (Fig. 2B4): A nerve hook is used to gently retract the nerve root. The axilla and shoulder regions of the nerve root are carefully explored, and the herniated disc material (nucleus pulposus) is removed to achieve complete release and mobilization of the nerve root.

We have incorporated these steps into the revised manuscript (highlighted in Track Changes). We hope this detailed explanation meets your requirements and perfectly illustrates the surgical procedure.

________________________________________

Reply to Reviewer #2:

Reviewer #2’s Comment: The authors have appropriately addressed all of the concerns raised in the prior review. The revisions have improved the accuracy and clarity of the manuscript, and no further comments are warranted.

Response:

Thank you very much for your time, expertise, and positive evaluation of our manuscript. Your constructive comments during the previous round of review have greatly improved the quality of our paper.

________________________________________

Reply to Reviewer #3:

Reviewer #3’s Comment: Overall, I believe the revisions satisfactorily address the prior concerns, and I have no further substantive comments.

Response:

We sincerely appreciate your thorough review and encouraging comments. We are glad that our revisions have met your expectations. Thank you for your valuable contribution to improving our study.

Attachment Submitted filename: Response to Reviewers.docx

# Editor report: Decision Letter 2

**DOI:** 10.1371/journal.pone.0359633.r005

**Authors:** Dean Chou

17 Sep 2026

Efficacy evaluation of the cervical posterior “keyhole technique” in the treatment of cervical radiculopathy: A retrospective case series

PONE-D-26-28826R2

Dear Dr. Zhou,

We’re pleased to inform you that your manuscript has been judged scientifically suitable for publication and will be formally accepted for publication once it meets all outstanding technical requirements.

Within one week, you’ll receive an e-mail detailing the required amendments. When these have been addressed, you’ll receive a formal acceptance letter and your manuscript will be scheduled for publication.

An invoice will be generated when your article is formally accepted. Please note, if your institution has a publishing partnership with PLOS and your article meets the relevant criteria, all or part of your publication costs will be covered. Please make sure your user information is up-to-date by logging into Editorial Manager at Editorial Manager® and clicking the ‘Update My Information' link at the top of the page. For questions related to billing, please contact billing support.

If your institution or institutions have a press office, please notify them about your upcoming paper to help maximize its impact. If they’ll be preparing press materials, please inform our press team as soon as possible -- no later than 48 hours after receiving the formal acceptance. Your manuscript will remain under strict press embargo until 2 pm Eastern Time on the date of publication. For more information, please contact onepress@plos.org.

Kind regards,

Dean Chou, MD

Academic Editor

PLOS One

Additional Editor Comments (optional):

Reviewers' comments:

# Editor report: Acceptance letter

**DOI:** 10.1371/journal.pone.0359633.r006

**Authors:** Dean Chou

PONE-D-26-28826R2

PLOS One

Dear Dr. Zhou,

I'm pleased to inform you that your manuscript has been deemed suitable for publication in PLOS One. Congratulations! Your manuscript is now being handed over to our production team.

At this stage, our production department will prepare your paper for publication. This includes ensuring the following:

* All references, tables, and figures are properly cited

* All relevant supporting information is included in the manuscript submission,

* There are no issues that prevent the paper from being properly typeset

You will receive further instructions from the production team, including instructions on how to review your proof when it is ready. Please keep in mind that we are working through a large volume of accepted articles, so please give us a few days to review your paper and let you know the next and final steps.

Lastly, if your institution or institutions have a press office, please let them know about your upcoming paper now to help maximize its impact. If they'll be preparing press materials, please inform our press team within the next 48 hours. Your manuscript will remain under strict press embargo until 2 pm Eastern Time on the date of publication. For more information, please contact onepress@plos.org.

You will receive an invoice from PLOS for your publication fee after your manuscript has reached the completed accept phase. If you receive an email requesting payment before acceptance or for any other service, this may be a phishing scheme. Learn how to identify phishing emails and protect your accounts at https://explore.plos.org/phishing.

If we can help with anything else, please email us at customercare@plos.org.

Thank you for submitting your work to PLOS One and supporting open access.

Kind regards,

PLOS One Editorial Office Staff

on behalf of

Dr. Dean Chou

Academic Editor

PLOS One
